# Identification and Interpretation of the Completely Oblique Rasch Bifactor Model

**DOI:** 10.1017/psy.2025.14

**Published:** 2025-04-24

**Authors:** Denis Federiakin, Mark R. Wilson

**Affiliations:** 1 Department of Economic Education, Johannes Gutenberg University of Mainz, Mainz, Germany; 2 Institute of Psychology, Goethe University Frankfurt, Frankfurt, Germany; 3 Centre for Psychometrics and Educational Measurement, Institute of Education, HSE University, Moscow, Russia; 4 Berkeley Evaluation and Assessment Research (BEAR) Center, Graduate School of Education, UC Berkeley, Berkeley, CA, USA

**Keywords:** bifactor models, item response theory, multidimensional random coefficients multinominal logit model, oblique bifactor models, Rasch models

## Abstract

Bifactor Item Response Theory (IRT) models are the usual option for modeling composite constructs. However, in application, researchers typically must assume that all dimensions of person parameter space are orthogonal. This can result in absurd model interpretations. We propose a new bifactor model—the Completely Oblique Rasch Bifactor (CORB) model—which allows for estimation of correlations between all dimensions. We discuss relations of this model to other oblique bifactor models and study the conditions for its identification in the dichotomous case. We analytically prove that this model is identified in the case that (a) at least one item loads solely on the general factor and no items are shared between any pair of specific factors (we call this the G-structure), or (b) if no items load solely on the general factor, but at least one item is shared between every pair of the specific factors (the S-structure). Using simulated and real data, we show that this model outperforms the other partially oblique bifactor models in terms of model fit because it corresponds to the more realistic assumptions about construct structure. We also discuss possible difficulties in the interpretation of the CORB model’s parameters using, by analogy, the “explaining away” phenomenon from Bayesian reasoning.

Bifactor models (Holzinger & Swineford, 1937) are a common approach in IRT for modeling composite constructs. These models enable the simultaneous estimation of a general factor, which is measured by all items, and specific factors, which are measured by subsets of them (see, for example, Figure 1). Bifactor models are particularly useful for capturing a general factor in tests with varied item types or in testlet-based assessments, where groups of items are linked by a common stimulus (Reise, 2012). They are also a popular focus in psychometric research because they generalize higher-order models mathematically (Gignac, 2016). Additionally, bifactor models have a constrained form known as the testlet model, which is equivalent to higher-order models (Rijmen, 2010).

**Figure 1:**
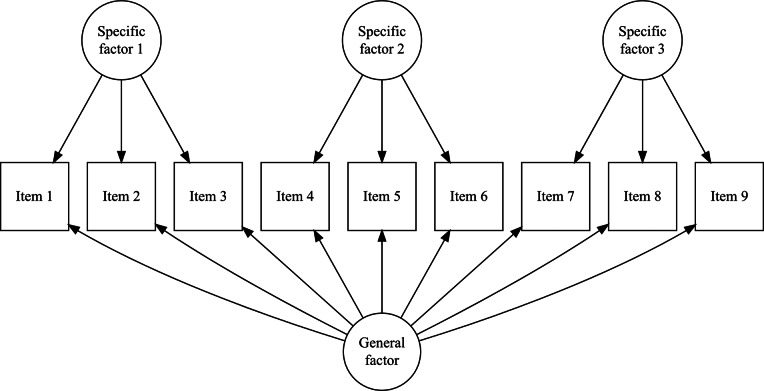
A bifactor structure with three specific factors. All items load on the general factors and on one specific factor.

Traditional bifactor models are constrained by a restrictive assumption: the general and specific factors must be orthogonal, meaning they are uncorrelated. According to the traditional framework, this assumption is necessary to ensure model identification (Reise et al., 2010). However, from an interpretational and substantive perspective, this assumption is often nonsensical despite its mathematical justification. For example, consider a bifactor model applied to a test comprising items that measure algebra and geometry to derive a general mathematics score. This approach requires assuming that the general mathematics factor is uncorrelated with both algebra and geometry scores. Additionally, it demands that algebra and geometry scores themselves be uncorrelated. Such assumptions make it challenging to interpret the resulting factor scores as meaningful representations of content domains (Wilson & Gochyyev, 2020).

Eid et al. (2017) highlighted a related paradox using stochastic measurement theory, demonstrating that orthogonal bifactor models should only be applied when the specific factors are interchangeable—essentially drawn at random from the universe of specific factors. This assumption, however, does not hold when specific factors represent distinct subject matter domains, such as algebra and geometry. Eid et al. (2017) further concluded that this requirement is rarely met in practice, leading to the overuse of bifactor models due to inappropriate measurement design.

To address this limitation, researchers often justify their use of bifactor models by aligning their application with specific modeling objectives. Frequently, the focus is on the general factor, with specific factors serving as a mathematical tool to account for local dependencies among items caused by shared content or stimuli (e.g., DeMars, 2013). Alternatively, some researchers emphasize the specific factors and view the general factor as a common source of error variance across all items (Hendy & Biderman, 2019). In such cases, the assumption of total orthogonality contradicts theoretical models of the construct.

Still, psychometricians often treat secondary factors as nuisance dimensions, enabling them to overlook interpretational challenges. However, this approach is suboptimal for modeling composite constructs, as it prioritizes mathematical convenience over an accurate representation of the relationships between components. Attempts have been made to differentiate the contexts in which orthogonal bifactor models are applied. For example, these models have been shown to perform exceptionally well in measurement contexts (Cai et al., 2011; Jeon et al., 2018; Wang & Zhang, 2019) but fail to yield reliable estimates in predictive contexts (Zhang et al., 2021; Zhang, Luo, Sun, et al., 2023).

To address these limitations, several extensions of bifactor IRT models have been proposed, providing partial solutions to the challenges of traditional bifactor models. These extensions allow for the direct estimation of specific entries in the variance–covariance matrix of the latent person parameter space. Notable examples include the Extended Rasch Testlet Model (ETM; Paek et al., 2009) and the Generalized Subdimensional Model (GSM; Brandt & Duckor, 2013), both of which have been developed within the Rasch modeling framework (Rasch, 1993).

The ETM permits the estimation of covariances between specific factors and the general factor while maintaining orthogonality among the specific factors. In contrast, the GSM enforces orthogonality between the general factor and the specific factors but allows correlations among the specific factors, albeit under complex constraints. More recently, partially oblique bifactor models, such as GSM (but without those constraints), have been shown to be analytically identified within the covariance structure modeling framework if the factor loading matrix satisfies certain stringent requirements (Fang et al., 2021). However, these models have demonstrated high numerical instability in practice (Zhang, Luo, Zhang, et al., 2023), leading researchers to advise caution in their use. Furthermore, none of these partially oblique bifactor models allow for the unrestricted estimation of the entire variance–covariance matrix. As a result, the interpretation of factor scores and correlations remains as challenging as it is in traditional bifactor models.

The purpose of this article is twofold. First, from a theoretical perspective, we introduce the CORB model within the confirmatory IRT paradigm. This model, with certain limitations, enables the direct estimation of all entries in the variance–covariance matrix of person parameters, simplifying the interpretation of model parameters. We explore the structure and interpretation of the CORB model in relation to existing oblique bifactor models. As a special case of the Multidimensional Random Coefficients Multinomial Logit Model (MRCMLM; Adams et al., 1997), the CORB model can be calibrated using dedicated software such as the ConQuest program (Adams et al., 2020), the TAM package for the R language (Robitzsch et al., 2025), or other tools for Generalized Linear Mixed Effect Modeling (e.g., de Boeck et al., 2011).

Second, this article makes a practical contribution by describing two specific test dimensionality structures that facilitate the estimation of all correlations among person parameters. The first structure involves having at least one item that does not load on any specific factor, effectively serving as an indicator for the general factor. The second structure requires that every pair of specific factors share at least one item. We demonstrate how these two structures ensure the identification of the CORB model and discuss their practical implications.

The article is organized as follows: First, we describe the MRCMLM framework and outline the conditions necessary for identifying multidimensional Rasch models derived from this framework. Second, we present the CORB model and examine the conditions under which it is identified. Third, we compare the CORB model with other oblique bifactor models. Fourth, we conduct a simulation study to demonstrate that the CORB model is more flexible and performs better than other oblique Rasch models in terms of technical characteristics. Fifth, we provide a real data example using a reading assessment for first-graders and discuss challenges in interpreting the CORB model. Finally, we conclude with a discussion of the CORB model and potential directions for future research and application.

## 1 MRCMLM framework

Assume a test consists of 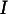 items ( 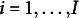 ), where each item has 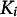 categories ( 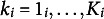 ), and the total number of categories in the test is 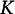 (so that 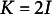 in the case of a dichotomous test). Without loss of generality, we assume all items are dichotomous. Consequently, each item is described by a single parameter ( 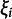 ), and the total number of item parameters, 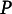 , equals the number of items, 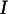 .

Further, let the test measure 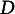 latent factors 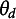 ( 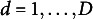 ). Each of 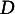 test scores, 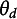 , is assumed to follow a distribution marginalized to have a mean ( 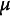 ) of zero for model identification (i.e., 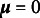 ). For simplicity, this distribution is assumed to be normal with an estimated variance 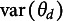 . However, this normality assumption is not necessary in the general case (Le & Adams, 2013). The latent space of person parameters is then defined by a multivariate normal distribution characterized by a vector of means 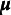 and a variance–covariance matrix 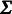 .

According to the reflective perspective on measurement, we assume a predetermined correspondence between every response category of each test item and a specific latent factor. This correspondence is governed by a scoring matrix 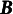 (explained below). The first category of every item is scored as zero, which serves to identify the model and establishes this category as the reference category.

Formally, the MRCMLM is expressed as follows:(1)

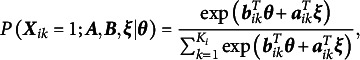

 where 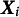 is a vector-valued random variable indicating 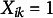 if a response to item 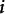 is in category 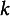 (out of all possible 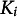 categories) and 0 otherwise,


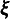
 is a vector of 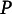 item parameters ( 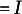 item difficulties in the dichotomous case),


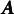
 is the design matrix ( 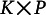 ), composed of design vectors 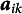 (each of length 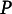 ),


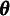
 is the vector of person parameters, representing a 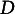 -dimensional latent space,


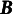
 is the scoring matrix ( 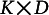 ), composed of scoring vectors 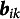 (each of length 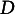 ).

The design matrix 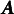 defines the relationships between item categories and item parameters, while the scoring matrix 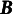 links item categories to the test dimensions. If non-zero entries of 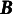 are estimated as free parameters, they are interpreted as discrimination (or scoring) parameters, and the model corresponds to the 2PL approach in IRT. Conversely, if these entries are constrained to unity, the model follows the Rasch approach.^1^ Generally, the 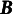 matrix is structured as a factor loading matrix. The MRCMLM framework encompasses a wide range of models, including multidimensional, dichotomous, and polytomous models (using the adjacent logit link function), as well as other specialized models from the exponential family, within both the Rasch and 2PL paradigms.

### 1.1 Volodin and Adams condition for Identifying a D-dimensional Rasch model

Volodin and Adams (2002) outlined the condition required for identifying multidimensional Rasch models with all correlated dimensions. They demonstrated that an oblique multidimensional Rasch model is identifiable if the following condition is met^2^:(2)

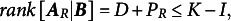

 where 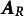 is a reduced design matrix, carefully constructed to preserve the original model’s structure, and 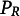 is the length of the reduced vector of item parameters corresponding to 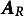 .

If Equation 2 holds, the model permits the direct estimation of all entries in the variance–covariance matrix. However, constructing 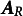 involves systematically dropping 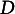 item parameters from 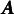 to impose the necessary constraints for identification. To establish this result, Volodin and Adams (2002) derived a series of theorems, which we reproduce and discuss in detail in this section. In Section 1.2, we provide a detailed illustration of this procedure for the test dimensionality structure from Figure 1.

For the dichotomous multidimensional model described in Equation 1, model identification is typically achieved by constraining the average ability in each dimension to 0 ( 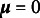 ). However, this constraint is not strictly necessary. In the general case, these averages can be estimated as part of the Rasch model, and the resulting vector of constants 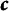 can then be subtracted from the corresponding item difficulties without altering the likelihood of the data:

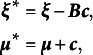

then,

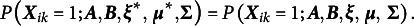



Naturally, the problem of model identification reduces to demonstrating that 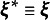 and 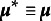 for any response profile 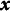 in a vector-valued variable 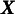 . In other words, if the model is identified, the matrices 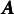 and 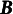 must satisfy the condition 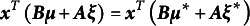 . Voloding and Adams propose a sequence of theorems to establish when this condition holds. To do so, they consider the vector 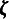 of length 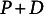 , which concatenates the vectors 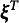 and 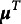 , and the corresponding vector 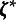 of the same length, which concatenates the vectors 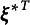 and 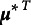 .

Theorem 1. The model (1) can only be identified if 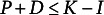 .

Proof. Assume, 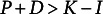 . Then, 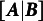 cannot be of full column rank, as it can have at most 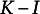 non-zero rows. Consequently, there would be no unique solution for the vector 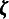 , contradicting the definition of 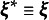 and 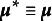 or an identified model (1). Therefore, 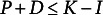 must hold.

Theorem 2. The model (1) can only be identified if 
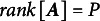
 , 
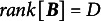
 , and 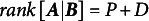 .

Proof. The matrix 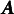 must conform to the vector 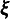 of length 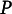 , it should have 
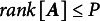
 . Assume, 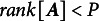 . In this case, 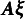 does not provide a unique solution for 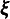 , and the model (1) cannot be identified. Therefore, if the model is identified, 
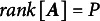
 must hold. Similarly, 
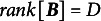
 must also hold. Consequently, 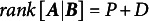 also must be true.

Theorem 3. The model (1) can be identified only if 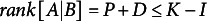 .

Proof. The necessary conditions directly follow from Theorems 1 and 2. To prove the sufficiency, consider the identification condition for model (1)

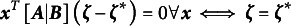
 .

The matrix 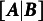 is of size 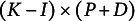 with 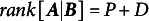 , and 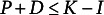 . Thus, it is possible to remove 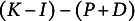 rows from 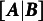 to construct a square submatrix of size 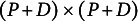 in full rank. Denote this matrix as 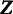 .

Let 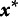 be a vector corresponding to 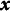 with the same elements removed as the rows excluded from 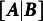 to construct 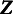 . To avoid trivial solutions, we constrain 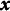 (and 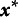 ) to not be entirely zero. Then, 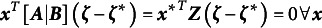 is equivalent to 
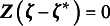
 . This holds *iff* 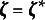 , meaning that 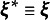 and 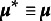 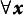 , which is the target of showing that the model is identified.

It follows that, in general, the special cases of model (1) are not identified unless the vector 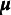 is not constrained to all zeros. Additionally, this procedure does not address the covariance matrix 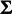 , which spans the latent space of person parameters. Instead, it emphasizes that item parameters play the central role in the identification of Rasch models. This procedure applies broadly to any completely oblique multidimensional Rasch model, including the CORB model.

However, in many scenarios, the constraint of 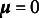 still might be insufficient for identification. For instance, if the test dimensionality structure aligns with that shown in Figure 1, the matrix 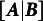 fails to satisfy the condition in Equation 2. More generally, avoiding the constraint 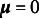 can be advantageous. In such cases, as noted in Theorem 3, the full matrix 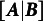 will not suffice for identification. This is where the construction of the reduced design matrix 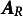 becomes essential.

After substituting 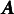 with 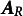 in 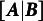 (resulting in 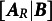 ), Theorem 3 can often be proven in the marginal case where 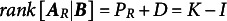 , as demonstrated in this article. The key question, then, is how to construct 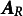 so that it fully preserves the structural properties of 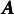 , up to a vector of additive constants 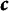 , while still enabling the proof of Theorem 3. The process of construction 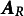 lies at the core of the Volodin–Adams procedure.

For the Volodin–Adams procedure 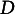 subsets of items ( 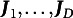 ) are defined, each of size 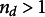 . It is not necessary that 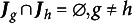 ; but it is necessary that 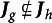 , 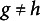 . Next, matrix 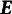 of size 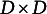 is constructed, where 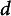 th row is represented by the vector 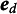 , consisting of the column sums of values in 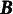 for the items in 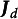 . Additionally, a set of 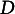 items, 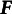 , is identified such that 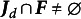 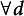 .

Theorem 4. If 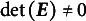 , then the completely oblique multidimensional dichotomous model can still be specified if 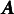 is substituted with 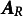 , where 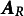 is reduced by 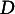 columns compared to 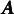 , such that 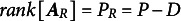 .

Proof. Assume, 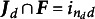 . Now, set 
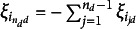
 , where 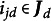 . Under this assumption, the 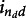 th row of 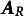 will contain value “−1” in the columns corresponding to 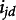 (*j* 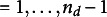 ), and “0” in the column corresponding to 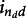 . Repeat this procedure 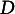 times, such that 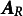 contains 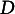 all-zero columns. Delete these all-zero columns, to obtain 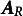 with 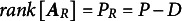 .

Now suppose 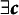 such that 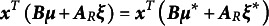 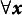 . For an extreme case, select 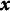 such that it contains values of “1” in positions 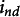 ( 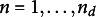 ) and “0” elsewhere. Then, 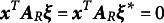 , and 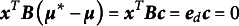 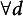 . Now if 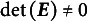 , then the only solution is 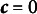 , which implies 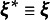 and 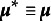 .

This completes the proof of Equation 2. Essentially, Volodin and Adams (2002) demonstrated that the matrix 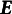 function as a scaling and rotation matrix for the vector 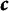 (of length 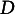 ), which consists of constants that can be added to the vector of means 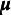 and subtracted from the item parameters in each corresponding dimension without affecting the overall likelihood of the data. If the determinant of 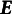 is non-zero, the vector 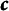 can only contain zeros, indicating that the model under the given 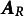 is identified. The challenge of constructing the matrix 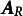 relies on a well-known result in the partial identification of Rasch models. According to this result, constraining the averages of latent dimensions to zero, or fixing one of the item parameters to zero, does not affect the relative rank order of items and respondents. Instead, it merely shifts the latent scale numerically, leaving the model’s interpretability and validity unaffected.

The full description of the procedure for the general case (including the polytomous case) is beyond the scope of this article; for further details, refer to Volodin and Adams (2002). The Supplementary Materials for this article include the R code for a function that automates this procedure in the dichotomous case.

### 1.2 *An Example of test dimensionality structure from Figure* ***1***

Using the Volodin and Adams procedure, it can be shown that if a test has a structure similar to the one presented in Figure 1, it is impossible to construct non-nested sets of item parameters with a non-zero determinant of 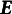 when all dimensions are oblique. For the structure in Figure 1 under a dichotomous test, 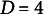 , 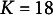 , 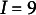 .

The complete design matrix 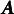 is of 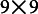 size, where each column corresponds to a single item difficulty parameter, and each row corresponds to a single item. Strictly speaking, in the design matrix 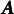 , each row should describe a single category for a single item, resulting in a matrix of 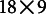 size. However, since all rows corresponding to zero categories are redundant (composed entirely of zeros), they can be excluded from the design matrix for simplicity. In this simplified representation, an entry of “0” in the matrix indicates that the corresponding parameter *is not* applied to the respective item category, and an entry of “1” indicates that the item parameter *is* applied. The resulting design matrix 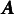 is as follows:(3)

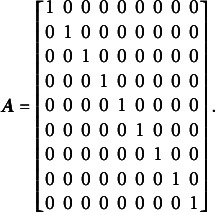



The scoring matrix 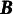 for Figure 1 is of 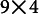 size (analogous to the design matrix 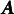 , it would typically be 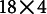 but, again, zero rows can be excluded for simplicity). Each row in 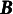 corresponds to a single item, and each column corresponds to a single latent factor. The general factor is represented by the first column. In this matrix, an entry of “0” indicates that the corresponding category *does not* load on the respective factor, and an entry of “1” indicates that the item *does* load on the respective factor. The resulting scoring matrix 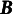 is as follows:(4)

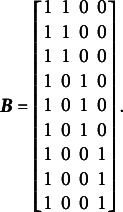



To construct the reduced design matrix 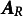 , define four sets of item parameters: (i) items 1 and 2, (ii) items 3 and 4, (iii) items 5 and 6, and (iv) items 7, 8, and 9. These sets correspond to the grouping indicated by the dashed lines in Equation 5, which illustrate how the item parameters are partitioned into subsets for constructing 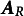 :(5)

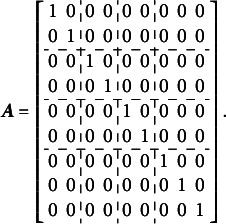



In this case, the reduced design matrix 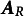 is defined as follows, with the length of the reduced vector of items parameters 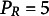 :(6)

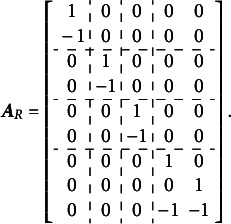



The corresponding partitioning of the matrix 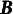 is(7)

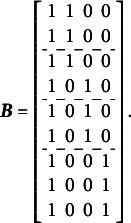



Then, the matrix 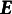 , consisting of the set-wise sums of the entries in 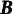 is given by(8)

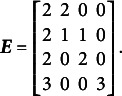



Consequently, 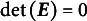 , which effectively terminates the Volodin–Adams procedure by showing that 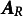 does not fully preserve the structure of the original model. As a result, 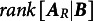 fails to satisfy Equation 2, confirming that the reduced design matrix does not enable the identification of the model:(9)

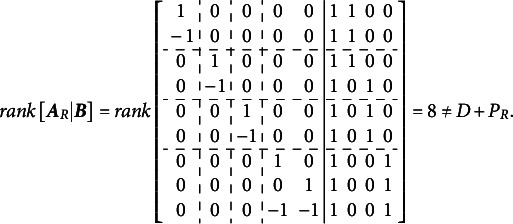



Repeating this procedure for any arbitrary partitioning of items into sets demonstrates that such a test dimensionality structure does not permit the identification of the oblique bifactor model. Consequently, constraining all covariances among person dimensions to zero becomes necessary for model identification, leading to the orthogonal bifactor Rasch model (Wang & Wilson, 2005).

However, it is important to note that this procedure describes an analytical approach to model identification. In practice, the general principles of modeling suggest that some constraints can be introduced into analytically unidentified models to achieve empirical identification (Kenny, 1979; Rindskopf, 1984). The Volodin–Adams procedure does not account for such constraints; it specifically evaluates whether the completely oblique multidimensional Rasch model is analytically identified.

The orthogonal Rasch bifactor model represents an extreme yet common solution for identifying bifactor models, where all factor covariances are simultaneously constrained to zero. In Section 3.3, we discuss that while this solution ensures identification, it may be overly restrictive and unsuitable for certain purposes.

## 2 The completely oblique Rasch bifactor model

The CORB model is distinguished from the orthogonal bifactor Rasch model by two key features.

### 2.1 Distinction 1: the variance–covariance matrix

The first distinction is that, unlike the orthogonal bifactor Rasch model, the CORB model enables the simultaneous estimation of all entries in the variance–covariance matrix 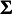 of latent factors. For example, in a test consisting of three specific factors, the variance–covariance matrix of the dimensions in the latent person parameter space for the CORB model takes the form shown in Equation 10. This contrasts with the orthogonal bifactor model, where the corresponding variance–covariance matrix is restricted as shown in Equation 11 (Wang & Wilson, 2005).(10)

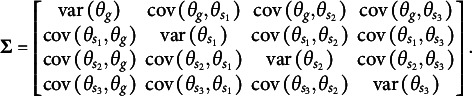


(11)

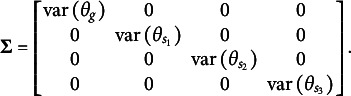



From the comparison of the variance–covariance matrices, it is evident that the orthogonal bifactor Rasch model is a special case of the CORB model. Specifically, constraining all off-diagonal elements in Equation 10 to zero results in the matrix form given in Equation 11.

### 2.2 Distinction 2: the structure of test dimensionality

The orthogonal bifactor Rasch model (Wang & Wilson, 2005), when specified for the structure of test dimensionality similar to Figure 1, can be expressed in scalar notation as follows^3^:(12)

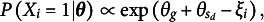

 where 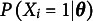 is the probability of a response of 1 to item 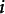 , given the vector-valued latent variable 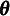 of dimensionality 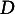 ,


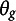
 is the value of the general factor,


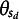
 is the value of the specific factor 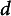 ( 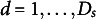 , where 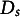 is the number of specific factors, so that 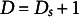 due to the general factor), and


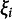
 is the difficulty of item 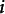 .

We refer to test dimensionality structures similar to Figure 1 as “clear bifactor structures”: no items load solely on the general factor without also loading on specific factors, and no specific factors share any items. Jennrich and Bentler (2012) describe such bifactor structures as “perfect cluster structures,” referring to item clustering logic.

**Figure 2:**
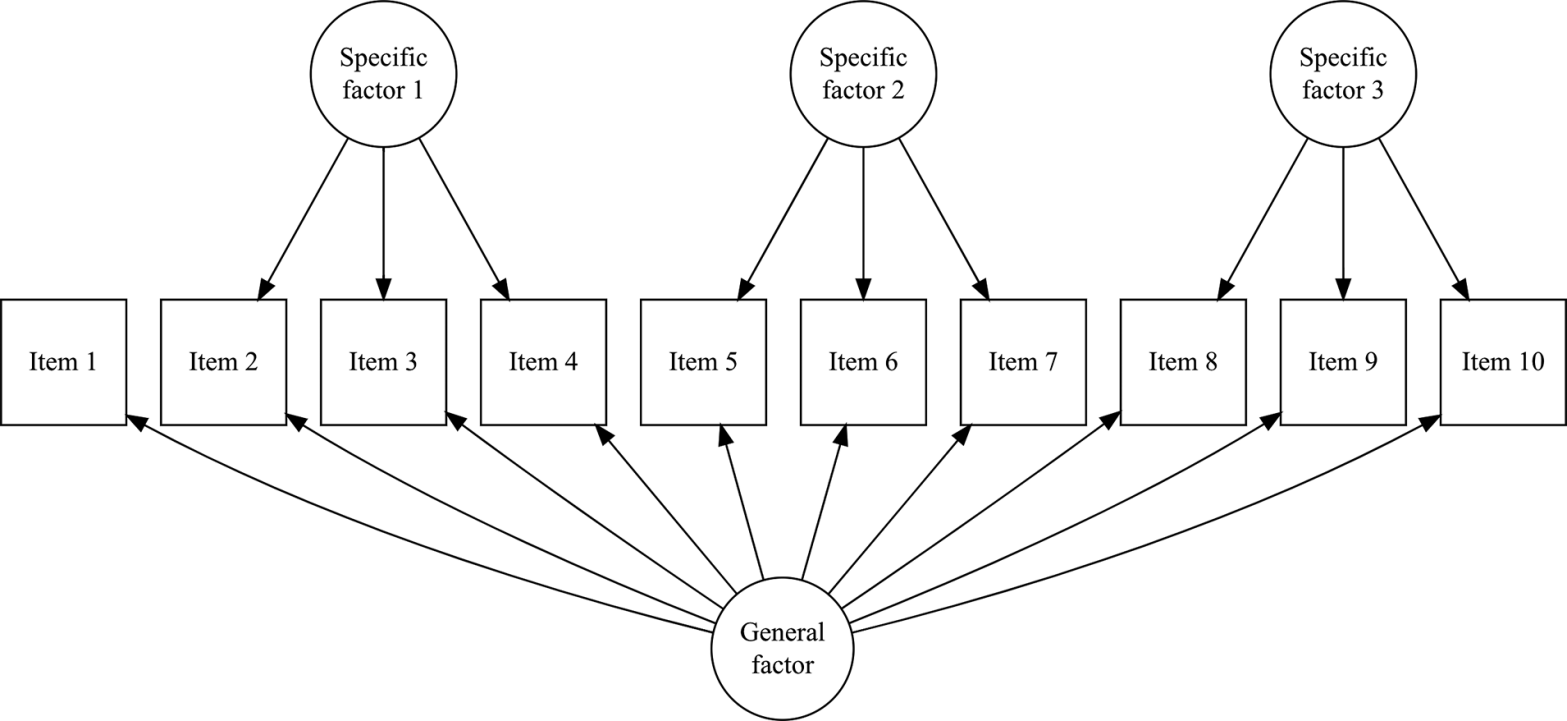
A bifactor structure for identifying the CORB model. Item 1 loads solely on the general factor, while no items are shared between any pair of specific factors. Factor covariances are non-zero but are not depicted in the figure.

The CORB model is not identified for all such clear bifactor structures. However, the CORB model becomes identifiable when the test dimensionality structure resembles the one shown in Figure 2—that is, when there is at least one item that loads on the general factor but not on any specific factor.

To define the test dimensionality structure for a case like Figure 2, researchers must identify two sets of items: 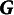 —the set of items (consisting of at least one item) that loads only on the general factor and not on any specific factor, and 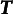 —the set of items that load on both the general factor and one specific factor.

The complete scalar formulation of this model is as follows:(13)

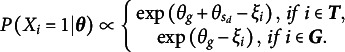



For the structure shown in Figure 2, 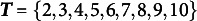 , representing items that load on both the general and specific factors, and 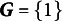 , representing the item that loads solely on the general factor. We call CORB models with such structures “G-structures.”

The implementation of the Volodin–Adams procedure, analogous to the outline provided for Equations 3–9, is illustrated below for the G-structure depicted in Figure 2. This implementation demonstrates that the procedure enables the construction of 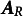 , where the corresponding 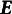 matrix has a non-zero determinant, thereby satisfying Equation 2.

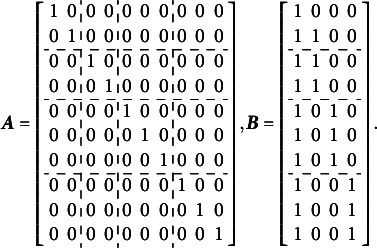




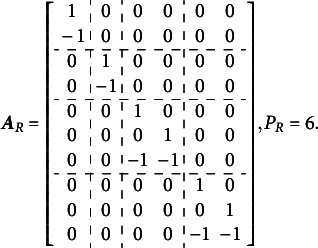




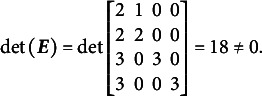




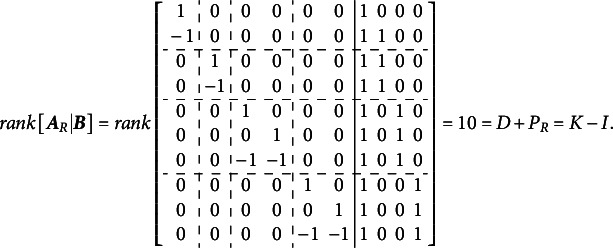



The intuitive explanation for this logic can be drawn from the work of Zhang, Luo, Zhang, et al. (2023). They demonstrated that the identification of partially oblique bifactor factor-analytical models hinges on the factor loadings matrix. In Rasch modeling, however, all discrimination parameters are constrained to unity. Now, consider a “clear,” completely oblique bifactor structure. As shown earlier (Equations 3–9), such a Rasch bifactor model is not identifiable. However, adding a “construct item” (from the 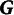 set) to this bifactor structure increases the number of observed variables without increasing the number of estimated factor loadings. This adjustment renders the model identifiable.

That said, the G-structure requires at least one item that loads solely on the general factor, effectively defining it. Eid et al. (2017) refer to such items as “reference indicators,” as all other indicators’ parameters are estimated relative to this reference. Including general construct items, however, may be impractical when the test is purely composite and comprises distinct components. While Eid et al. (2017) emphasize the necessity of such items and Zhang, Luo, Zhang, et al. (2023) provide detailed guidance on selecting them (including real-world examples, which interested readers may consult in their work). Overall, however, this requirement poses a challenge for test developers and item writers.

**Figure 3:**
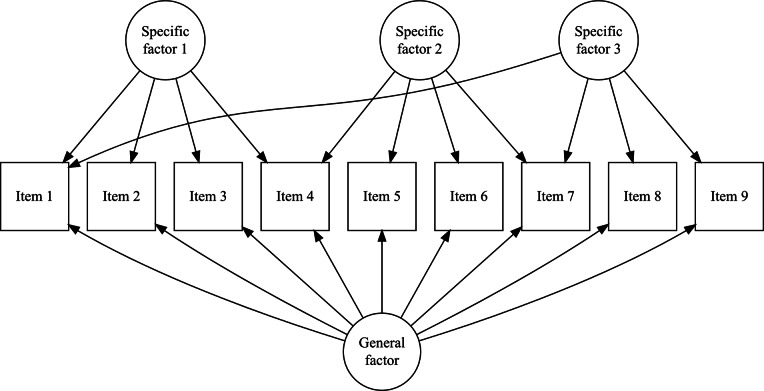
A bifactor structure for identifying the CORB model. No single item loads solely on the general factor, but at least one item is shared between each pair of specific factors (i.e., item 1 for specific factors 1 and 3, item 4 for specific factors 1 and 2, and item 7 for specific factors 2 and 3). Factor covariances are non-zero but are not depicted in the figure.

Fortunately, an alternative test dimensionality structure can identify the CORB model, as shown in Figure 3. This structure requires that every pair of dimensions share at least one item.

To specify the structure of test dimensionality for a case similar to Figure 3, one must define two sets of items: 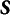 —the set of items that load on two specific factors, and 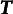 —the set of items that load on one specific factor.

The complete scalar formulation of this model is given as(14)

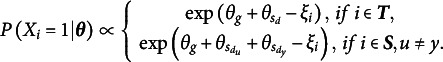



For the structure depicted in Figure 3: 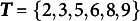 , representing items that load on the general factor and one specific factor, and 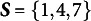 , representing items that load on the general factor and two specific factors. We call CORB models with such structures “S-structures.”

The calculations below illustrate the implementation of the Volodin and Adams procedure for the test dimensionality structure shown in Figure 3:

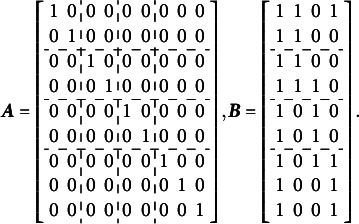




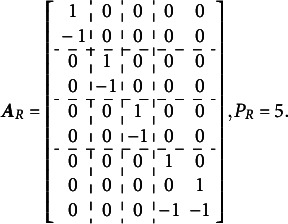




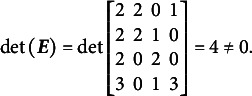




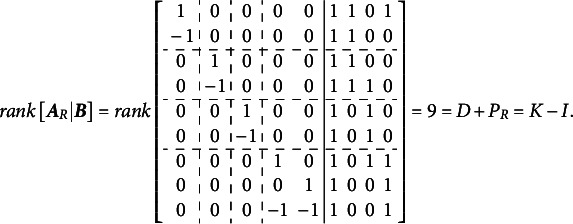



To intuitively understand why the S-structure allows for CORB model identification, we turn to the geometric interpretation of the multidimensional Item Characteristic Surface (ICS; Ackerman, 1994; Reckase & McKinley, 1991). In this framework, the shape of the multidimensional ICS is determined by two key factors: (1) the angle between the latent dimensions (represented as the arccosine of the Pearson correlation between the factors) measured by the items with within-item multidimensionality, and (2) the discrimination parameters of these items on the respective factors.

In Rasch models, however, the discrimination parameters are constrained to unity. As a result, the structure of item response variance in the shared items directly defines the correlations between the latent factors, since the discriminations cannot vary freely. This fixed discrimination ensures that shared items play a critical role in establishing the relationships among the latent dimensions, making the S-structure effective for CORB model identification.

When a test consists of multiple content areas, the S-structure of test dimensionality may offer a more practical approach to CORB model identification. To specify this structure, a test developer can enhance the existing test by adding new items that combine, in a compensatory manner, pairs of specific factors. Alternatively, Bifactor Exploratory Structural Equation Modeling (Morin et al., 2016) can aid in identifying items suitable for inclusion in the 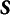 set. Such items should exhibit significant and relatively similar factor loadings to the “main” items associated with the specific factors. This approach is often more feasible than creating “construct items” to define the general factor, which can be challenging in most testing contexts.

It is crucial to note that not all items are suitable to serve as “construct items” in G-structures or “shared items” in S-structures. A defining feature of Rasch modeling is that all items with the same dimensionality structure share the same discrimination parameters. This concept, when related to the logic of factor analysis, implies that all items with the same dimensionality structure allocate the same proportions of response variance to the different latent factors.

For example, if a “shared item” in an S-structure has a distribution of response variance across latent factors that does not align with the variance–covariance structure of other items loading on these factors, it is likely to be flagged as a misfitting item in item fit analyses. Similarly, “construct items” in G-structures are subject to the same requirement. As a result, modifying the dimensionality structure of existing test items or developing new tests identifying the CORB model remains a challenging task.

In both cases, deviations from the clear bifactor structure (Figure 1) are necessary to identify the CORB model. However, it is important to note that the G-structure and S-structure do not exhaust the possible dimensionality structures capable of identifying the CORB model. To determine whether the CORB model is identifiable for a particular test structure, it is necessary to apply the Volodin-Adams procedure.

Additionally, both the G-structure and S-structure also identify the orthogonal Rasch bifactor model. This is because the orthogonal Rasch bifactor model is a special case of the more general CORB model, which is identifiable under these structures. In such cases, instead of being defined solely by Equation 12, the orthogonal Rasch bifactor model would also be described by Equations 13 or 14, depending on the structure.

## 3 Other oblique bifactor models

### 3.1 The Extended Rasch Testlet model

The closest relative of the CORB model in the literature is the Extended Rasch Testlet model (ETM; Paek et al., 2009). The ETM allows for the estimation of non-zero correlations between the specific factors and the general factor while maintaining orthogonality among the specific factors. The variance–covariance matrix 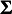 of person parameters for the same number of dimensions as in Equations 10 and 11 is represented as follows:(15)

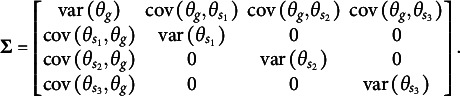



The scalar specification of the ETM follows the same form as Equations 13 or 14. The only difference between the CORB model and the ETM lies in their variance–covariance structures. Specifically, the ETM is a special case of the CORB model: constraining all off-diagonal elements in Equation 10, except for those corresponding to the covariances between the general factor and specific factors (i.e., the first row and the first column), results in Equation 15. This implies that the same test dimensionality structures that identify the CORB model also identify the ETM. Furthermore, in the original paper (Paek et al., 2009), the G-structure of the ETM was used for model identification, corresponding to Equation 13, as several “construct items” were included.

At the same time, the original orthogonal Rasch bifactor model (originally called the Rasch Testlet Model or RTM; Wang & Wilson, 2005) is a special case of the ETM. Constraining *all* off-diagonal elements in Equation 15 to zero results in Equation 11, which represents the variance–covariance matrix of the RTM. Consequently, these models form a hierarchy of nested models, enabling their comparison using a likelihood ratio test.

### 3.2 The Subdimensional family of models

The GSM (Brandt, Duckor, 2013) and the Subdimensional Rasch Model (SRM; Brandt, 2008) allow for the estimation of correlations between specific factors while maintaining orthogonality between the specific factors and the general factor. To achieve this, these models require the exclusion of one specific factor from estimation:(16)

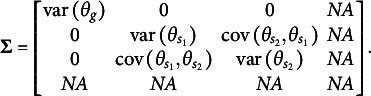



In this setup, the last specific factor in Equation 16 is defined as the negative sum of all remaining specific factors. This constraint necessitates a modification of the scoring matrix 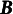 , as shown in Equation 17. Below is an example of the scoring matrix 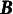 for a clear bifactor structure (Figure 1):(17)

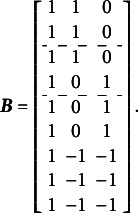



Comparing Equation 17 with Equation 4 shows that this modification of the scoring matrix 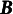 makes the GSM *not a special case of the CORB model*, as it does not simply constrain some parameters to zero.

Due to the exclusion of a specific factor, it is necessary to recalibrate the GSM with alternative reparameterizations at least three times to obtain the complete variance–covariance matrix of the specific factors (i.e., GSM and SRM require 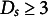 ). This process involves:

(1) Excluding the last specific factor ( 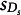 ) to recover all covariances between specific except those involving last specific factor ( 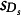 ), as described by Equations 16 and 17.

(2) Excluding the second to last specific factor ( 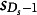 ) to recover all covariances involving the last specific factor ( 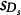 ), except for the covariance between specific factors 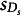 and 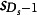 . This step results in(18)

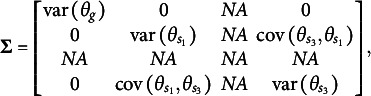


(19)

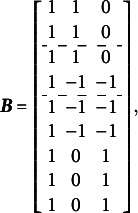



(3) Excluding the third to last specific factor ( 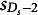 ) to recover the covariance of the specific factors 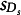 and 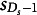 . This step results in(20)

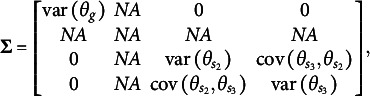


(21)

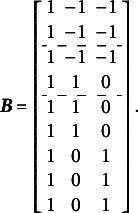



These different reparameterizations describe the same latent space of person parameters, differing only in which parameters are directly estimated. This is possible because all reparameterizations satisfy the constraint 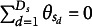 for every respondent. The choice of which factor to exclude is arbitrary and does not affect model fit. This can also be verified using the Volodin–Adams procedure across different reparameterizations.

Equation 22 applies to all reparameterizations since they differ only in the scoring matrix 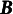 :(22)

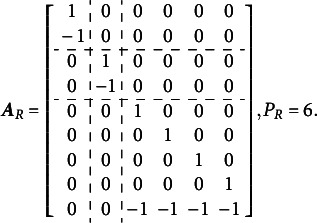



Equations 23–25 demonstrate that the determinants of the 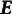 matrices are non-zero when the partitioning specified in Equation 22 is applied to Equations 17, 19, and 21:(23)

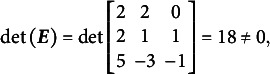


(24)

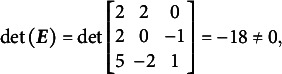


(25)

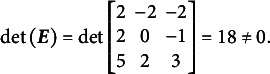



The absolute values of all determinants are identical, indicating that not only does 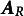 fully specify the model represented by 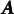 , but also that the same partitioning of the 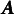 matrix across different parameterizations of the same GSM model results in the same latent person parameter space. This property arises from the nature of the 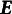 matrix, which moderates the scaling and rotation of the constants vector 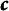 . Equation 26 confirms that all reparameterizations of the GSM model are identifiable.(26)

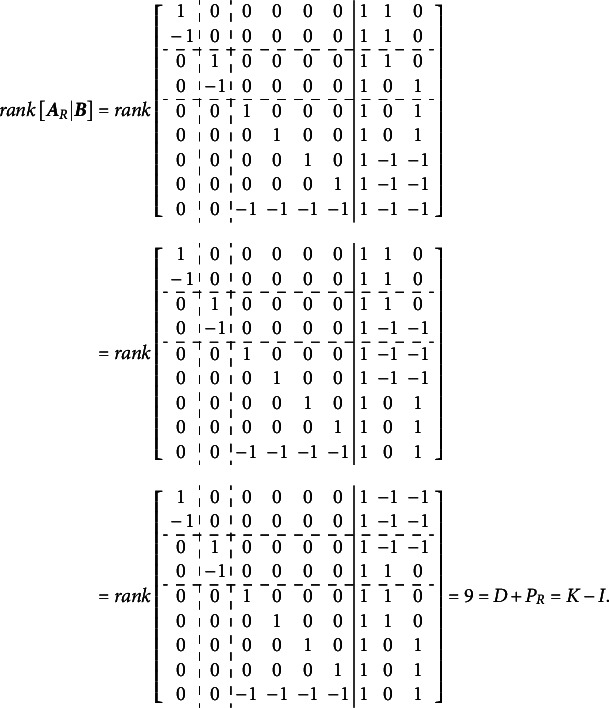



Moreover, empirical comparisons of parameters estimated multiple times across different reparameterizations demonstrate that they converge to the same values (Federiakin, 2020).

From the model definitions described above, it follows that calibrating the GSM to study the variance–covariance matrix becomes meaningless when the number of specific factors is two. In such cases, one of the two specific factors will always be excluded from calibration under any parameterization, and their correlation will necessarily be constrained to −1, since the sum of the specific factors is fixed to zero for every respondent.

Unlike the ETM and the CORB model, the GSM can be identified in cases of clear bifactor structures. The GSM follows the scalar form:(27)

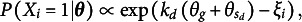

 or equivalently:(28)

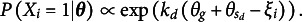



The parameter 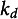 distinguishes the GSM from the SRM (which follows Equation 12) and highlights that the GSM is not a special case of the CORB model. The parameter 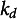 is essential for addressing an implicit assumption in the SRM, which assumes equality of variances across all specific factors. Consequently, the GSM requires an additional constraint of 
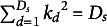
 . The notation in Equation 27 was initially proposed by Brandt and Duckor (2013), while the notation in Equation 28 was introduced later by Robitzsch et al. (2025, p. 145) for simplicity in estimation. It is important to note, however, that these two notations are equivalent and both align with the Rasch modeling paradigm.

Additionally, the GSM differs from the ETM and the CORB model in its interpretation of the latent parameter space. In the GSM, the specific factors are orthogonal to the general factor, and their sum is constrained to zero. As a result, the GSM models the relationships among the components *within the general factor* (see Brandt, 2017, for the algebraic formalization). The construct components themselves, under this unidimensional interpretation, are represented as the sums of the corresponding specific factors and the general factor. In contrast, the ETM and, by extension, the CORB model describe components that are *additional* to the general factor. In this sense, the GSM is conceptually closer to a unidimensional model, while the ETM and CORB models are “more multidimensional” in their interpretation.

Consequently, the GSM does not belong to the model hierarchy of RT-ETM-CORB. Comparisons between the GSM and these models can only be conducted using information criteria such as AIC (Akaike, 1974) and BIC (Schwarz, 1978). These criteria penalize model fit for additional parameters (AIC) and adjust for sample size (BIC).

### 3.3 Other possibilities for oblique bifactor modeling

It is important to note that the models discussed so far do not represent the full range of oblique bifactor models. Exploratory bifactor factor analysis offers additional oblique bifactor solutions. For example, Jennrich and Bentler (2012) proposed two criteria for bifactor rotation of the factor loading matrix. However, their approach has an approximating nature and comes with additional requirements.

First, their method constrains the specific factors to be orthogonal to the general factor, making its interpretation the reverse of the ETM. Second, their approach is not identified in clear bifactor cases. When the data structure is truly bifactor, Jennrich and Bentler’s criteria fail to provide a unique factor solution. Finally, this approach belongs to the exploratory data analysis paradigm, which poses challenges for its application in hypothesis testing, modeling growth and change, or conducting measurement invariance analysis. As a result, the practical application of these models in testing scenarios remains limited.

Lorenzo-Seva and Ferrando (2019) proposed a somewhat similar logic for partially oblique exploratory bifactor modeling. Their approach involves a sequence of rotation steps designed to build upon one another, stabilizing the results of their procedure.

Partially oblique confirmatory bifactor models have recently gained attention in the field of factor analysis. Fang et al. (2021) demonstrated that, within the covariance structure model (applicable to both identity and probit link functions), it is not analytically necessary for bifactor models to have orthogonal specific factors if the factor loading matrix satisfies certain conditions of linear independence. A key condition for their identification is the linear independence of columns in the submatrices of the factor loadings matrix. Specifically, if the submatrices corresponding to the specific factors have a column rank of at least 2, models with correlated specific factors can be identified (for details, see Fang et al., 2021).

In their work, Fang et al. (2021) adapted the general results of Anderson and Rubin (1956) and the conclusions of Grayson and Marsh (1994) for Multitrait-Multimethod (MTMM) models to bifactor models. However, more recently, Zhang, Luo, Zhang, et al. (2023) revealed that these models are highly numerically unstable in practice, highlighting the need for more rigorous investigation into their empirical identifiability and cautioning against their unchecked use. Interestingly, Zhang et al. (2021) and Zhang, Luo, Zhang, et al. (2023) also proposed a model augmentation approach equivalent to the G-structures of test dimensionality described in this article. They demonstrated that this approach stabilizes estimation algorithms and resolves many convergence issues in the case of freely estimated factor loadings. Notably, these suggestions follow the structure of partially oblique bifactor models—specifically Bifactor-(S-1) and Bifactor-(S*I-1) models with correlated specific factors (Eid et al., 2017)—which have been critically discussed by Koch and Eid (2024).

In the context of this article, these findings suggest that while there are structural parallels between factor analysis and logistic IRT, the identification strategies can differ significantly (Bee et al., 2023). Further exploration of these differences and their implications for model stability and practical application remains a promising area for future research.

Overall, the Bifactor-(S-1) and Bifactor-(S*I-1) models (Eid et al., 2017), the augmentation approach by Zhang et al. (2021) and Zhang, Luo, Zhang, et al. (2023), and G-structures all fit within a common structural framework. However, by fixing factor loadings to known values, researchers are able to estimate correlations among all latent factors. Crucially, this alters the interpretation of these correlations. In traditional partially oblique bifactor models (such as Bifactor-(S-1) or Bifactor-(SI-1)), correlations between specific factors are partial correlations—conditional on the general factor—similar to the correlations between general and specific factors in the ETM model. In contrast, in the CORB model the latent dimensions are not treated as residuals; they are not conditioned on one another. As a result, their variances are not strictly separated, allowing for a more holistic interpretation of the latent structure.

Additionally, the literature describes other CORBs that impose specific constraints on the variance–covariance matrix of person parameters. For example, Robitzsch et al. (2025) introduce models with a zero constraint on the sum of covariances across all dimensions (Robitzsch et al., 2025, p. 143), or a zero constraint on the sum of variances and covariances of all dimensions (Robitzsch et al., 2025, pp. 143–144). These models appear to be identifiable under clear bifactor structures, though this conclusion does not directly follow from the Volodin–Adams procedure.

This suggests that certain constraints on the variance–covariance matrix can render analytically unidentified multidimensional Rasch models empirically identifiable. Consequently, some special cases of the CORB model—such as the ETM—may also be empirically identified under clear bifactor structures.

In contrast, the G-structures and S-structures of test dimensionality described in this paper provide analytical (in this context—definitive) identification for the CORB model and all its special cases, including the ETM. However, the models introduced by Robitzsch et al. (2025) have only been described in the software literature and have not yet been thoroughly studied. Moreover, their practical interpretation remains unclear, as it is nearly impossible to align such constraints with realistic expectations from the data or the structure of the construct being measured.

Finally, a wide range of longitudinal and MTMM models are relevant to this type of bifactor modeling. Specifically, within the longitudinal framework, derivations of Jöreskog’s (1970) simplex model (Wilson et al., 2012) can be viewed as nested bifactor models with G-structures. These models produce latent estimates of difference scores that reflect changes in ability across measurement occasions. This is conceptually similar to bifactor models in which specific factor estimates represent the difference between the general ability and the ability required to solve the items associated with a given specific factor.

While longitudinal models can estimate the full correlation matrix of latent dimensions—thanks to constraints placed on the factor loadings of anchor items (Duncan & Duncan, 2004)—the reliability of the resulting difference scores has been a longstanding concern (e.g., Cronbach & Furby, 1970). Although the debate on the reliability of factor scores continues (see Trafimow, 2015), we explore this issue in the context of the CORB model through our simulation study.

Several special cases of MTMM models are also highly relevant to partially oblique bifactor models. In particular, some MTMM models adopt a latent difference score approach by imposing constraints on factor loadings (e.g., Pohl et al., 2008). Other models have modified these constraints so that specific factors do not reflect the difference between two abilities but rather the deviation from a person-specific average across all specific abilities—resulting in latent mean models (e.g., Pohl & Steyer, 2010).

More broadly, a growing body of research is investigating the conditions under which correlation matrices in these models are identifiable (see Bee et al., 2023, for a recent review). These modeling approaches have been extended to a variety of applications, ranging from survey validation to rater assessments (Eid et al., 2024), and now represent one of the most prominent and rapidly evolving areas in psychometrics.

## 4 The simulation study

### 4.1 Design

We conducted a simulation study to examine the recovery of model parameters by the CORB model and compare it to existing partially oblique bifactor models. For simplicity in comparing model fits, the simulations utilized only the G-structure of test dimensionality. The study addressed three Research Questions related to parameter recovery:

RQ1: How does the number of “construct items” affect parameter recovery?

RQ2: How does the number of specific factors affect parameter recovery?

RQ3: How does the number of items per specific factor affect parameter recovery?

To address the research questions:

1. For RQ1, we varied the number of construct items from 1 to 2 to 3, while keeping the number of items per specific factor and the number of specific factors constant (5 and 3, respectively).
2. For RQ2, we varied the number of specific factors from 3 to 4 to 5, while keeping the number of items per specific factor and the number of construct items constant (3 and 1, respectively).
3. For RQ3, we varied the number of items per specific factor from 3 to 5 to 7, while keeping the number of specific factors and the number of construct items constant (5 and 3, respectively).

Overall, we designed 9 simulation conditions, with 100 replications for each condition. In each replication, we calibrated the CORB model, the ETM, three reparameterizations of the GSM (averaging the results across them), and the orthogonal RTM, all using the same test dimensionality structures for comparison.

In the replications of these conditions, we randomly varied the variance–covariance matrices of the latent person parameter space, ensuring they were positive-definite. The variances ranged from 0.3 to 4 logits, with all dimensions (including the general factor) being oblique, reflecting a realistic setup. Across all simulations, the sample size was fixed at 2,000, and the item difficulties were spaced equally from −2 to 2 logits. Items were assigned alternating loads on specific factors, though the number of items varied.

To compare the simulation results, we utilized the following metrics:

- AIC and BIC indices: To assess model fit while accounting for parameter complexity and sample size.
- Pearson correlation: Between Expected a Posteriori (EAP; Bock & Mislevy, 1982) ability estimates and their true values.
- EAP reliabilities: To assess the consistency of EAP estimates (Adams, 2005).
- Root Mean Squared Error (RMSE) of the factor correlation matrix was estimated as the Root Mean Squared Frobenius norm of the difference matrix between estimated and true covariance matrices across all replications, providing inherent normalization to its values and robustness to the varying covariance scale:

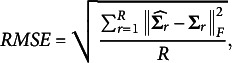

 where 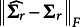 is the Frobenius norm of the difference matrix between the true covariance matrix 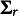 in replication 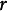 and the estimated covariance matrix 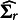 ,


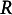
 is the total number of replications.

- Bias in the variance estimates:

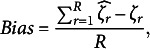

 where 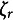 is a true value of the parameter in the replication 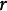 , 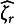 is an estimate of the parameter in the replication 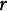 .

Since RMSE was essentially estimated in the correlation matrix, it does not account for potential biases in the variance estimates of latent dimensions. Bias in the variances was used to account for this limitation. It allows us to evaluate the general tendency of a model to overestimate or underestimate the variances of latent dimensions, as well as the expected magnitude of this over- or underestimation.

First, we expect the CORB model to yield the most accurate parameter estimates compared to other partially oblique bifactor models, since it was used as the data-generating model and reflects the most realistic assumptions about the construct structure. Specifically, we anticipate that the CORB model will recover the most accurate correlation estimates. Also, we expect the CORB model to demonstrate the best global model-data fit across all conditions.

Second, we expect that the number of specific factors and the number of construct items will have the greatest impact on model fit, as these elements directly influence the dimensionality of the test structure. Therefore, in RQs 1 and 2, we expect the CORB model to outperform the other oblique bifactor models most significantly.

Third, we expect the CORB model to achieve the highest EAP reliabilities of test scores. This is because EAP estimation can incorporate information from the variance–covariance matrix of the dimensions, allowing scores in the oblique multidimensional model to “reinforce” one another in proportion to their correlations (de la Torre & Patz, 2005).

For the simulations we used TAM package v. 3.7-16 for R software (Robitzsch et al., 2021).

### 4.2 Results

#### 4.2.1 RQ1: how does the number of “construct items” affect parameter recovery?

The ETM failed to converge in 23% of cases when there was 1 construct item, 19% of cases with 2 construct items, and 22% of cases with 3 construct items. These results suggest that the number of construct items does not significantly impact the ETM’s convergence behavior. In contrast, all other models converged 100% of the time, regardless of the number of construct items. This indicates potential distortions in the person parameter space during estimation, rendering the ETM difficult or impossible to estimate consistently (Table 1).

**Table 1:** Comparison of the bifactor models of interest for the first research question of the simulation study

Statistics	Number of construct items	Model			
		RT	ETM	GSM	CORB
		0.754	0.758	0.771	0.813
	1	(0.145)	(0.153)	(0.137)	(0.104)
		0.776	0.788	0.787	0.827
	2	(0.145)	(0.131)	(0.139)	(0.099)
		0.806	0.832	0.803	0.848
Average correlation of the general factor EAP point estimates with their true values (SD)	3	(0.135)	(0.099)	(0.154)	(0.087)
		0.591	0.607	0.542	0.705
	1	(0.164)	(0.176)	(0.196)	(0.137)
		(0.150)	(0.154)	(0.187)	(0.120)
	2	0.607	0.636	0.563	0.725
		0.605	0.637	0.553	0.733
Average correlation of specific factors EAP point-estimates with their true values (SD)	3	(0.152)	(0.146)	(0.199)	(0.101)
		0.615	0.696	0.789	0.698
	1	(0.211)	(0.140)	(0.102)	(0.129)
		0.625	0.725	0.783	0.695
	2	(0.215)	(0.130)	(0.122)	(0.148)
		0.660	0.750	0.789	0.721
Average EAP reliability of the general factor (SD)	3	(0.206)	(0.130)	(0.132)	(0.139)
		0.401	0.472	0.502	0.531
	1	(0.178)	(0.158)	(0.141)	(0.143)
		0.414	0.479	0.511	0.544
	2	(0.163)	(0.147)	(0.136)	(0.129)
		0.411	0.475	0.493	0.539
Average EAP reliability of the specific factors (SD)	3	(0.168)	(0.142)	(0.152)	(0.128)
		32538.81	32298.78	32264.23	32209.76
	1	(1257.91)	(1275.37)	(1245.66)	(1252.28)
		34178.04	33869.69	33878.67	33779.92
	2	(1429.97)	(1351.05)	(1355.31)	(1356.78)
		36444.85	36133.05	36153.31	36002.29
Average AIC (SD)	3	(1679.01)	(1617.01)	(1627.57)	(1619.58)
		32650.83	32427.60	32387.45	32355.39
	1	(1257.91)	(1275.37)	(1245.66)	(1252.28)
		34295.66	34004.11	34007.49	33931.14
	2	(1429.97)	(1351.05)	(1355.31)	(1356.78)
		36568.07	36273.07	36287.74	36159.12
Average BIC (SD)	3	(1679.01)	(1617.01)	(1627.57)	(1619.58)
		0.377	0.303	0.445	0.154
	1	(0.080)	(0.112)	(0.110)	(0.081)
		0.379	0.293	0.435	0.121
	2	(0.089)	(0.115)	(0.168)	(0.056)
		0.390	0.297	0.430	0.092
Average RMSE of correlation matrix (SD)	3	(0.090)	(0.104)	(0.138)	(0.049)
		−0.043	0.062	0.658	−0.138
	1	(1.170)	(0.755)	(1.138)	(0.526)
		−0.030	0.085	0.518	−0.093
	2	(1.094)	(0.588)	(1.078)	(0.390)
		−0.120	−0.017	0.339	−0.030
Average bias of the general factor variance (SD)	3	(1.008)	(0.531)	(0.979)	(0.283)
		−0.170	−0.169	−0.614	−0.166
	1	(1.234)	(1.124)	(1.210)	(0.463)
		−0.202	−0.224	−0.603	−0.104
	2	(1.083)	(1.042)	(1.403)	(0.325)
		−0.224	−0.260	−0.546	−0.030
Average bias of the specific factor variances (SD)	3	(0.890)	(0.862)	(1.540)	(0.281)

In general, the results indicate that the CORB model outperforms other partially oblique bifactor models in terms of the correlation of parameter estimates with their true values and global model fit. Across all simulation conditions, the CORB model consistently provides better parameter recovery. Interestingly, however, the reliability of the general factor in the CORB model is lower than that of the GSM. This aligns with the fact that the GSM primarily focuses on a unidimensional interpretation of the test, thereby forcing more information from item scores into the general factor.

Similarly, the general factor reliability in the ETM is higher than in the CORB model for a related reason: the ETM’s orthogonality assumption between specific factors enhances the general factor’s reliability while weakening the specific factor reliability compared to the CORB model. As expected, the worst performance overall is observed for the completely orthogonal bifactor model, both in terms of reliability and model fit.

When comparing the RMSE of the correlation matrix, the results align with expectations for the RTM, the ETM, and the CORB model: the more general the model, the better it recovers correlations. However, the GSM exhibits a surprising result. Despite fitting better than the ETM according to AIC and BIC, the GSM produces a latent space of person dimensions that deviates the most from the data-generating space. This outcome reflects the GSM’s modeling approach, which constrains the components of the construct to lie within the general factor rather than treating them as additional to it.

Interestingly, the RTM, the ETM, and the GSM show minimal systematic bias in variance estimates (relative to the standard deviation of this bias). In contrast, the CORB model tends to slightly underestimate the variances of both the general and specific factors, particularly when the test includes only 1 construct item. Nevertheless, the CORB model demonstrates superior stability in terms of the bias-variance trade-off compared to other models.

Regarding RQ1, increasing the number of construct items improves the performance of all models. This global improvement can likely be attributed to test length—a well-established factor in improving the precision of parameter estimates, as longer tests provide more data upon which parameter estimates are based.

#### 4.2.2 RQ2: how does the number of specific factors affect parameter recovery?

The ETM failed to converge in 8% of cases for 3 specific factors, 20% of cases for 4 specific factors, and 32% of cases for 5 specific factors. Unlike the results in the previous simulation (RQ1), this suggests that the ETM’s convergence is influenced by the complexity of the latent person parameter space, with higher numbers of specific factors leading to greater convergence issues. In contrast, all other models converged 100% of the time, regardless of the number of specific factors (Table 2).

**Table 2:** Comparison of the bifactor models of interest for the second research question of the simulation study

Statistics	Number of specific factors	Model			
		RTM	ETM	GSM	CORB
		0.695	0.688	0.710	0.750
	3	(0.194)	(0.180)	(0.186)	(0.147)
		0.767	0.768	0.771	0.795
	4	(0.135)	(0.143)	(0.130)	(0.115)
		0.790	0.779	0.796	0.814
Average correlation of the general factor EAP point estimates with their true values (SD)	5	(0.131)	(0.148)	(0.130)	(0.110)
		0.524	0.532	0.504	0.643
	3	(0.164)	(0.200)	(0.188)	(0.144)
		0.383	0.385	0.527	0.472
	4	(0.328)	(0.348)	(0.172)	(0.384)
		0.304	0.324	0.558	0.375
Average correlation of specific factors EAP point-estimates with their true values (SD)	5	(0.350)	(0.349)	(0.168)	(0.425)
		0.517	0.601	0.673	0.601
	3	(0.239)	(0.180)	(0.157)	(0.171)
		0.626	0.694	0.747	0.690
	4	(0.186)	(0.122)	(0.114)	(0.131)
		0.650	0.685	0.767	0.713
Average EAP reliability of the general factor (SD)	5	(0.193)	(0.162)	(0.125)	(0.147)
		0.320	0.378	0.395	0.466
	3	(0.151)	(0.142)	(0.132)	(0.130)
		0.321	0.377	0.412	0.461
	4	(0.144)	(0.138)	(0.120)	(0.123)
		0.338	0.379	0.439	0.482
Average EAP reliability of the specific factors (SD)	5	(0.146)	(0.121)	(0.119)	(0.113)
		20256.51	20150.09	20095.42	20059.69
	3	(691.54)	(671.80)	(668.94)	(670.87)
		26347.71	26166.26	26028.61	25997.71
	4	(689.87)	(654.67)	(653.10)	(651.39)
		32190.70	32043.03	31744.09	31726.26
Average AIC (SD)	5	(1042.07)	(1068.81)	(986.44)	(992.03)
		20334.93	20245.30	20185.03	20171.71
	3	(691.54)	(671.80)	(668.94)	(670.87)
		26448.52	26289.48	26157.44	26154.53
	4	(689.87)	(654.67)	(653.10)	(651.39)
		32313.92	32194.26	31917.72	31933.49
Average BIC (SD)	5	(1042.07)	(1068.81)	(986.44)	(992.03)
		0.392	0.343	0.423	0.170
	3	(0.075)	(0.097)	(0.134)	(0.093)
		0.364	0.315	0.380	0.169
	4	(0.071)	(0.072)	(0.134)	(0.077)
		0.339	0.295	0.322	0.158
Average RMSE of correlation matrix (SD)	5	(0.051)	(0.052)	(0.087)	(0.055)
		−0.073	0.082	0.503	−0.191
	3	(1.046)	(0.787)	(1.055)	(0.541)
		0.037	0.074	0.574	−0.078
	4	(0.843)	(0.623)	(0.837)	(0.450)
		0.056	0.053	0.548	−0.130
Average bias of the general factor variance (SD)	5	(0.685)	(0.452)	(0.679)	(0.400)
		−0.203	−0.253	−0.662	−0.186
	3	(1.224)	(1.245)	(1.341)	(0.548)
		−0.192	−0.374	−0.316	−0.151
	4	(1.081)	(1.023)	(1.554)	(0.529)
		−0.102	−0.285	−0.085	−0.071
Average bias of the specific factor variances (SD)	5	(1.064)	(0.945)	(1.705)	(0.524)

In general, the results are consistent with the previous simulation and indicate that the CORB model outperforms the other partially oblique bifactor models across all key statistics—from the correlation between parameter estimates and their true values to global model fit (with the exception of the general factor reliability in the GSM model). The insights from the previous simulation study are repeated here: the GSM tends to recover the most reliable general factor scores, but this comes at the expense of interpreting the specific factors.

The CORB model provides a balance between the reliability of the general factor and the specific factors. It improves the reliability of the general factor compared to the traditional orthogonal bifactor model while simultaneously recovering the most reliable scores for the specific factors. As expected, the CORB model recovers the correlation matrix more accurately than all other bifactor models and remains significantly more stable in terms of variance estimates.

Interestingly, while increasing the number of specific factors reduces the underestimation of the general factor variance on average, the CORB model’s recovery of the general factor variance, although improved and more stable, does not surpass that of its special cases (such as the ETM or GSM). This may indicate that the CORB model requires special convergence criteria or longer estimation times to achieve better performance in complex test structures.

Regarding RQ2, increasing the number of specific factors tends to improve overall model performance for all models. However, as in RQ1, this improvement may primarily result from the increased test length, which enhances parameter precision by providing more data for estimation.

#### 4.2.3 RQ3: how does the number of items per specific factor affect parameter recovery?

The ETM failed to converge in 19% of cases with 3 items per specific factor, 41% of cases with 5 items per specific factor, and 66% of cases with 7 items per specific factor. These results indicate that the convergence of the ETM strongly depends on the length of the testlet, with longer testlets significantly reducing its likelihood of convergence. In contrast, all other models converged 100% of the time, regardless of the number of items per specific factor (Table 3).

**Table 3:** Comparison of the bifactor models of interest for the third research question of the simulation study

Statistics	Length of specific factors	Model			
		RTM	ETM	GSM	CORB
		0.850	0.846	0.850	0.868
	3 items	(0.083)	(0.084)	(0.084)	(0.071)
		0.865	0.866	0.862	0.893
	5 items	(0.098)	(0.126)	(0.101)	(0.064)
		0.849	0.862	0.849	0.894
Average correlation of the general factor EAP point estimates with their true values (SD)	7 items	(0.133)	(0.153)	(0.132)	(0.072)
		0.307	0.332	0.546	0.384
	3 items	(0.361)	(0.371)	(0.179)	(0.438)
		0.349	0.360	0.624	0.421
	5 items	(0.411)	(0.405)	(0.159)	(0.462)
		0.383	0.401	0.673	0.454
Average correlation of specific factors EAP point-estimates with their true values (SD)	7 items	(0.430)	(0.417)	(0.148)	(0.479)
		0.740	0.769	0.813	0.768
	3 items	(0.128)	(0.090)	(0.090)	(0.095)
		0.774	0.804	0.863	0.803
	5 items	(0.118)	(0.078)	(0.066)	(0.102)
		0.771	0.820	0.879	0.815
Average EAP reliability of the general factor (SD)	7 items	(0.152)	(0.096)	(0.089)	(0.106)
		0.339	0.392	0.430	0.493
	3 items	(0.138)	(0.118)	(0.113)	(0.109)
		0.432	0.471	0.547	0.569
	5 items	(0.154)	(0.133)	(0.119)	(0.114)
		0.508	0.531	0.633	0.642
Average EAP reliability of the specific factors (SD)	7 items	(0.163)	(0.148)	(0.116)	(0.113)
		36133.58	35988.58	35664.30	35568.71
	3 items	(1398.77)	(1337.36)	(1315.10)	(1323.55)
		55848.24	55656.65	55099.90	54986.73
	5 items	(2064.83)	(2088.40)	(2016.15)	(1994.92)
		74980.44	74311.80	73853.91	73716.20
Average AIC (SD)	7 items	(3645.04)	(3527.21)	(3575.12)	(3570.18)
		36268.00	36151.01	35849.13	35787.14
	3 items	(1398.77)	(1337.36)	(1315.10)	(1323.55)
		56038.68	55875.08	55340.73	55261.18
	5 items	(2064.83)	(2088.40)	(2016.15)	(1994.92)
		75226.88	74586.25	74150.75	74046.65
Average BIC (SD)	7 items	(3645.04)	(3527.21)	(3575.12)	(3570.18)
		0.343	0.295	0.347	0.101
	3 items	(0.054)	(0.055)	(0.149)	(0.040)
		0.339	0.277	0.311	0.093
	5 items	(0.051)	(0.051)	(0.084)	(0.033)
		0.344	0.281	0.319	0.089
Average RMSE of correlation matrix (SD)	7 items	(0.051)	(0.053)	(0.094)	(0.042)
		0.073	0.011	0.408	−0.077
	3 items	(0.649)	(0.429)	(0.661)	(0.260)
		0	−0.052	0.387	−0.093
	5 items	(0.541)	(0.332)	(0.541)	(0.254)
		0.009	−0.079	0.457	−0.126
Average bias of the general factor variance (SD)	7 items	(0.672)	(0.290)	(0.735)	(0.280)
		−0.204	−0.455	−0.276	0.025
	3 items	(1.002)	(0.867)	(1.780)	(0.411)
		−0.155	−0.143	−0.153	−0.014
	5 items	(0.723)	(0.652)	(1.618)	(0.280)
		−0.189	−0.094	−0.094	−0.060
Average bias of the specific factor variances (SD)	7 items	(0.787)	(0.654)	(2.431)	(0.284)

In general, the results are consistent with the previous simulations and once again demonstrate that the CORB model outperforms other partially oblique bifactor models across all simulation conditions and key statistics (with the exception of the general factor reliability in the GSM model). While the GSM model consistently yields the highest reliabilities, the CORB model produces the most accurate parameter estimates, as evidenced by the lower average RMSE of the correlation matrix and, in this case, also by the lower bias in variance estimates. Both increasing the number of “construct items” and lengthening the specific factors positively impact parameter recovery across all models.

Notably, while the GSM model consistently recovers a latent space that is furthest from the data-generating space, it paradoxically exhibits better model fit than the orthogonal bifactor model and the ETM, though not better than the CORB model. This highlights a critical limitation: a naïve comparison of the GSM with other models based solely on global model fit indices (such as AIC and BIC) can lead to substantial distortion in the interpretation of test scores. Such distortion undermines the intended construct validity that test developers aim for when designing the test. Therefore, we strongly recommend exercising caution when using the GSM model alongside the orthogonal bifactor model, the ETM, and the CORB model, as the GSM is fundamentally different from these models. Crude comparisons may result in significant validity threats.

Regarding RQ3, we can again conclude that, in general, the longer the test, the better the results, across all models.

## 5 A real data example

### 5.1 The test and the data

For the real data example, we used data from a low-stakes computerized assessment of reading literacy in Russian called “START.” This test is designed to measure first-graders’ reading literacy, defined as their ability to: (1) recognize letters of the Russian alphabet, (2) read words aloud, (3) read a short story aloud (“mechanical” reading), and (4) comprehend reading material (Ivanova & Kardanova-Biryukova, 2019). The assessment is conducted by teachers, who assist each student by opening the test in an internet browser and determining whether the student’s responses to each item are correct. All teachers follow standardized test administration guidelines provided by the test developers. The test consists of 35 dichotomous items, divided into four subsections based on the construct definition: letter recognition (9 items), reading words aloud (9 items), mechanical reading (3 items), reading comprehension (14 items).

For the sake of illustration, we calibrated the models without using a specific factor for reading comprehension. This approach forces the models to rely solely on the G-structure of the test dimensionality. Initially, this simplification was necessary to identify the CORB model and the ETM. To ensure consistency in model comparison, we applied the same G-structure to the RTM and GSM models. Consequently, all reading comprehension items were treated as “construct items” defining the general factor across all models. For the GSM, this meant that the 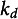 parameter was not estimated for the “construct items,” constraining their discrimination to unity.

This G-structure aligns with the construct definition, as reading literacy is conceptualized as the ability to comprehend texts. In this framework, the “lower-order” skills (letter recognition, word reading aloud, and mechanical reading) are considered prerequisites for reading comprehension.

The data was collected in November 2020 from a region in the Russian Federation. The sample includes 1,000 first-grade students, though it is not representative of the broader population.

### 5.2 Results

The results of the model comparison are presented in Table 4.

The correlation matrix from the ETM is presented in Table 5.

**Table 4:** The results of the model comparison of the real data

	Statistics	Model					
		RTM	ETM	GSM			CORB
				Variant 1	Variant 2	Variant 3	
	Deviance	20,667.8	20,413.2	21,394.5	21,392.9	21,388.2	20,320.7
	Number of parameters	39	42	42	42	42	45
	Sample size	1,000					
	AIC	20,745.8	20,497.2	21,478.5	21,476.9	21,472.2	20,410.7
	BIC	20,937.2	20,703.3	21,684.6	21,683.0	21,678.3	20,631.5
Reliability	General factor	0.920	0.922	0.942	0.942	0.943	0.914
	Letter recognition	0.466	0.620	–	0.391	0.395	0.619
	Reading of words	0.346	0.348	0.355	–	0.358	0.416
	Mechanical reading	0.390	0.520	0.422	0.404	–	0.539
Variance	General factor	8.740	9.207	10.019	9.878	10.160	9.199
	Letter recognition	6.168	5.678	–	4.223	4.588	5.809
	Reading of words	4.934	5.144	3.104	–	3.342	5.682
	Mechanical reading	5.101	6.778	3.098	2.814	–	6.987
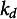	Letter recognition	–	–	0.315	0.350	0.330	–
	Reading of words	–	–	0.477	0.452	0.467	–
	Mechanical reading	–	–	1.635	1.635	1.635	–

*Note.* The results of the GSM are presented for 3 reparameterizations of it.

Likelihood Ratio Test confirmed that ETM fits better than RTM ( 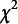 =254.6, df = 3, *p*-value <0.001).

Likelihood Ratio Test confirmed that CORB model fits better than both RTM ( 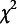 =347.1, df = 6, *p*-value <0.001) and ETM ( 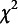 =92.5, df = 3, *p*-value <0.001).

**Table 5:** The correlation matrix from the ETM

	General factor	Letter recognition	Reading of words	Mechanical reading
General factor	1	-0.643	0.076	0.427
Letter recognition	-0.643	1	0	0
Reading of words	0.076	0	1	0
Mechanical reading	0.427	0	0	1

The gathered correlation matrix from the GSM is presented in Table 6.

**Table 6:** The gathered correlation matrix from all three reparameterizations of the GSM

	General factor	Letter recognition	Reading of words	Mechanical reading
General factor	1	0	0	0
Letter recognition	0	1	-0.579	-0.439
Reading of words	0	-0.579	1	-0.021
Mechanical reading	0	-0.439	-0.021	1

The correlation matrix from the CORB model is presented in Table 7.

**Table 7:** The correlation matrix from the CORB model

	General factor	Letter recognition	Reading of words	Mechanical reading
General factor	1	-0.650	0.043	0.390
Letter recognition	-0.650	1	0.280	0.011
Reading of words	0.043	0.280	1	0.579
Mechanical reading	0.390	0.011	0.579	1

### 5.3 Interpretation of results

The results from the real data application indicate that the CORB model fits the data better than other oblique bifactor models and the orthogonal bifactor model, which is expected since the CORB model is more general. However, the most significant distinction of the CORB model lies in its interpretability. Unlike other bifactor models, the CORB model allows for the direct interpretation of specific factors as “components” of general reading skills, as it permits these factors to correlate freely. In contrast, the assumptions of complete or partial orthogonality in other bifactor models imply that the extracted factor scores are abstract constructs, statistically “purified” from the influence of other factors.

The variances of all latent factors appear relatively high compared to similar studies. This can be attributed to the high “guttmanization” of students’ response profiles (Maggino, 2014) and the data collection conditions. Guttmanization likely results from the theoretical framework of the test, which presupposes a hierarchical structure of behavior indicators. In such a framework, a student is unlikely to answer a subsequent item correctly if they have already failed a preceding one. Additionally, on the practical side, the teacher (acting as the proctor) may end the testing session prematurely when a student begins to struggle, reinforcing the hierarchical nature of the responses. These factors likely increase item discriminations, and as a result, constraining discriminations to unity leads to relatively high variance estimates.

The estimates from different reparameterizations of the GSM exhibit some numerical fluctuations but tend to converge toward consistent values (albeit slightly less consistently than in previous studies; Federiakin, 2020).

One of the most challenging results to interpret is the occurrence of negative correlations in the correlation matrices. For example, a naive interpretation of Tables 5 and 7 might suggest that students who excel in letter recognition tend to struggle with reading comprehension, and vice versa. This apparent paradox affects both the ETM and CORB models. In the GSM case, the negative correlations in Table 6 can be explained from a technical standpoint: since the sum of specific dimensions is constrained to zero for each student, increasing one specific factor necessarily decreases the others, thereby inducing negative correlations.

Although the paradox of negative correlations appears puzzling from a content perspective, it is a well-documented phenomenon in within-item multidimensional models (van Rijn & Rijmen, 2012). This effect is known as the “explaining away phenomenon,” extensively studied within the framework of Bayesian reasoning and the causal interpretation of IRT models (Marsman et al., 2018). The principle behind this phenomenon is that “the confirmation of one cause of an observed event reduces the need to invoke alternative causes” (Wellman & Henrion, 1993, p. 287). In the context of within-item multidimensional IRT models, this implies that when an item loads on two latent dimensions in a compensatory manner, a student can succeed in answering the item through three possible scenarios:

1. Compensating for low ability on dimension 1 by having high ability on dimension 2.
2. Compensating low ability on dimension 2 by having high ability on dimension 1.
3. Having high ability on both dimensions.

However, scenario 3 is less likely, as it requires more conditions to be simultaneously satisfied. Therefore, negative correlations between dimensions arise because scenarios 1 and 2 dominate in the sample. Hooker and Finkelman (2010) proved this result for bifactor models that do not comply with the Schmid and Leiman (1957) constraints. Later, van der Linden (2012) provided a rigorous generalization of this result, while van Rijn and Rijmen (2012) graphically demonstrated it for all compensatory within-item multidimensional models.

Consequently, the negative correlations observed in the ETM and CORB models do not imply that students who are better at reading are worse at recognizing letters, or vice versa. Instead, these negative correlations are statistical artifacts resulting from the conditioning of parameter estimates on the distribution of student abilities. Therefore, this paradoxical outcome does not require extensive content interpretation or explanations based on substantial issues with the construct.

Interestingly, in both the ETM and CORB models, the correlations of the three specific factors with the general factor support the theoretical hierarchy of reading skills proposed by Ivanova and Kardanova-Biryukova (2019). Specifically, the closer a specific factor is to reading comprehension (which defines the general factor) in the theoretical hierarchy of skills, the stronger its correlation with the general factor becomes. It is important to note that the hierarchy of skills in this context is defined purely in terms of theoretical interpretation and does not impose structural constraints on the model itself. That is, although students are theoretically expected to acquire skills in a sequential manner, the model treats all skills as independent but correlated dimensions.

As a result, the closer two skills are in terms of cognitive content (e.g., letter recognition is cognitively closer to word reading than to mechanical reading), the stronger their correlation becomes. This effect may act as a counterbalance to the explaining-away phenomenon, driven by the similarity of the cognitive content across latent dimensions.

## 6 Discussion

Bifactor models are prevalent in psychometric literature because they directly extract the general factor from a truly composite test structure while accounting for local item dependence. However, they are notoriously difficult to interpret, as their identification requires highly restrictive constraints on the variance–covariance matrix. Specifically, the assumption of total orthogonality often results in models where only the general factor is practically interpretable, while the specific factors are typically treated as nuisance dimensions and ignored.

In response to these limitations, several partially oblique bifactor models have been proposed and studied. Notably, the ETM allows for direct estimation of correlations between the general factor and specific factors while maintaining orthogonality among the specific factors. Another well-documented model is the GSM, which allows correlations between specific factors but constrains them to be orthogonal to the general factor. However, the theoretical interpretations of these models vary considerably, as different constraints on the variance–covariance matrix lead to different conceptualizations of the construct being measured. Additionally, other bifactor models similar to the GSM—but without such constraints—can apparently be identified if the factor loading matrix satisfies specific conditions. Despite this, the interpretation and practical application of these models are often as complicated as those of orthogonal bifactor models due to the complexity of their underlying assumptions.

The purpose of this article was twofold:

1. To introduce the CORB model, which enables the direct estimation of all correlations between latent factors.
2. To describe the structures of test dimensionality that allow for the CORB model’s identification.

Through simulation studies and a real data example, we demonstrated that the CORB model outperforms other bifactor models in terms of model fit and the recovery of factor correlations. However, successful identification of the CORB model requires a specific test design structure. In this article, we introduced and analyzed two such structures:

1. G-structure (Figure 2): This structure requires that the test contain at least one “construct item” that loads solely on the general factor.
2. S-structure (Figure 3): This structure requires that no items load solely on the general factor, but at least one item is shared between every pair of specific factors.

These test dimensionality structures allow for direct estimation of all correlations between specific factors, simplifying the interpretation of the latent person parameter space. To analytically establish the identification of the CORB model, we applied the Volodin–Adams procedure, which verifies the identification of oblique Rasch models by examining the rank and structure of the design and scoring matrices.

However, as a within-item multidimensional compensatory IRT model, the CORB model is susceptible to paradoxical results, where two latent factors that are theoretically expected to correlate positively may instead be estimated as negatively correlated. For example, in the real data application, the specific factor “Letters recognition” was negatively correlated with the general factor, interpreted as reading comprehension. Nevertheless, such results are not truly paradoxical; they can be explained by the “explaining away” phenomenon from the Bayesian reasoning paradigm. From this perspective, these results are merely statistical artifacts that do not require extensive content interpretation.

Broadly, this aricle addresses the topic of bifactor model identification. Most researchers, particularly applied researchers and test developers, tend to assume that bifactor models must be orthogonal. In certain contexts, such as testlets and item bundles, this assumption is appropriate. Moreover, orthogonality significantly accelerates parameter estimation, as it prevents these models from falling victim to the “curse of dimensionality,” which exponentially increases computational complexity and slows down numerical integration as the number of correlated latent dimensions grows (Rijmen, 2009). In such cases, factors secondary to the researcher’s primary interest are often treated as nuisance dimensions that explain common variance across items.

However, our work demonstrates that deliberate modifications to the structure of test dimensionality can enable researchers to estimate all entries in the variance–covariance matrix of a bifactor model. This approach allows for models that align more closely with theoretical assumptions about the construct’s structure, particularly when the construct is intentionally composite rather than being artificially defined by the stimuli. Although such models are more computationally demanding and less advantageous from a technical standpoint due to their vulnerability to the curse of dimensionality, they offer significant theoretical benefits. Specifically, they are more useful in cases where researchers seek to explore the nuances of the construct structure (especially the correlation matrix of person dimensions) or apply the model in predictive measurement contexts (Zhang et al., 2021; Zhang, Luo, Sun, et al., 2023).

Furthermore, recent advances in parameterizing IRT (Converse, 2021) and factor-analytical models (Urban & Bauer, 2021) as artificial neural networks may help mitigate these computational challenges. Neural networks are far less susceptible—if not entirely immune—to the curse of dimensionality (Cheridito et al., 2021). Therefore, parameterizing the CORB model as a neural network could potentially eliminate computational inefficiencies, rendering computational time a negligible concern.

In the context of model identification, our article highlights that the conditions for identifying oblique bifactor models remain an area for further research. Notably, existing models that impose zero constraints on the sum of covariances or on the sum of variances and covariances of person parameters suggest that many oblique bifactor models that are analytically unidentified (under currently known procedures) may, in fact, be empirically identified. Further exploration of identification conditions could pave the way for the development of new oblique bifactor models with practical and theoretically meaningful interpretations.

Additionally, while models with freely estimated discrimination parameters require linear independence of factor loadings on the general and specific factors for identification (Fang et al., 2021; Zhang, Luo, Zhang, et al., 2023), this requirement appears irrelevant for Rasch models. In Rasch models, discrimination parameters are constrained to unity by definition, resulting in linearly dependent “factor loadings” on the general and specific factors. This distinction underscores the need for a more detailed investigation into the identification conditions specific to Rasch-based bifactor models.

Interestingly, since the ETM is a special case of the CORB model, it is conceptually possible to extend the CORB framework by proposing the Subdimensional Oblique Rasch Bifactor (SORB) model. The SORB model shares conceptual similarities with the GSM and is closely related to partially oblique models in factor analysis (Fang et al., 2021; Zhang, Luo, Zhang, et al., 2023), while also being a special case of the CORB model. Consequently, the SORB model follows the same identification requirements as the CORB model, since it adheres to the general formulations of Equations 13 or 14, similar to the ETM. However, instead of recovering the variance–covariance matrix in Equation 10, the SORB model recovers the matrix given by Equation 29:(29)

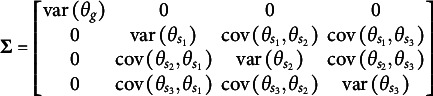



The constraint of correlations between specific factors and the general factor to zero, combined with the free estimation of correlations among specific factors, results in a “reversed” ETM, conceptually similar to the oblique bifactor solutions proposed by Jennrich and Bentler (2012) and Lorenzo-Seva and Ferrando (2019), but approached from a confirmatory modeling paradigm. This constraint also makes the orthogonal RTM a special case of both the ETM and the SORB model, though without nesting these models within one another.

This model is closer in interpretation to the ETM and CORB models than to the GSM. Specifically, it models specific factors as additional to the general factor, rather than as components of the general factor, as in the GSM. However, unlike the partially oblique bifactor models from factor analysis (Fang et al., 2021; Zhang, Luo, Zhang, et al., 2023), it constrains discrimination parameters, potentially making it easier to identify and more numerically stable. Further exploration of this model and its comparison to the GSM could be a valuable area for future research. In particular, a multi-step estimation procedure involving the following steps may improve numerical stability of parameter estimates and allow for estimation of the 2PL counterparts of all models used in this paper:

1. Preliminary estimation of a model.
2. Extraction of the estimated correlation matrix of the multivariate ability distribution from the preliminary estimation.
3. Fixing the correlation matrix in subsequent bifactor models with free factor loadings.
4. Estimating discrimination parameters given the fixed correlation matrix.

This approach could yield further improved model fit, more stable parameter estimates, and enhanced robustness of bifactor model applications.

This article has several limitations. First, we only considered dichotomous items. To identify the CORB model in a test with polytomous items, at least one category of at least one item must load solely on the general factor, or alternatively, at least one category of at least one item must be shared between every pair of specific factors. However, this result is specific to the adjacent logit link function. Extending these findings to other link functions, such as probit or cumulative link functions, represents a promising avenue for future research.

Moreover, the proposed structures of test dimensionality reflect a complex but tractable process of test development, particularly under partial credit scoring in educational assessments. Among the two structures discussed in this paper, developing items for the S-structure appears more feasible. For example, consider a test with a clear bifactor structure, where one subdimension represents addition skills and another represents subtraction skills. In this scenario, a researcher could create items requiring both skills (e.g., word problems combining addition and subtraction) to transform a clear bifactor structure into an S-structure.

Conversely, developing items that measure only general arithmetic skills (i.e., not specific to addition or subtraction) is likely more challenging. This example illustrates that while transforming an existing test into an S-structure may be achievable, constructing items for a G-structure, which requires purely general items, can be considerably more difficult.

Additionally, this paper does not delve into several important applied aspects of the CORB model. For example, we do not explore how the CORB model relates to the intricate connections between second-order models and bifactor models defined by the Schmid–Leiman constraints (Gignac, 2016; Mansolf & Reise, 2017; Rijmen, 2010). Furthermore, we do not address the item development process in detail and only briefly touch upon the topic of item fit.

A more in-depth discussion on the interpretation of the ETM and other partially oblique bifactor models is also necessary. Currently, their detailed interpretation remains unclear—particularly regarding when and how such complex variance–covariance matrix constraints can be expected to align with the underlying construct. Additionally, this article does not examine the potential impact of the CORB model on subscore reporting (Haberman et al., 2024). While the demand for interpretable scores on specific factors motivates our work, further research is needed to assess the added value of subscores derived from the CORB model.

Moreover, the CORB model is presented solely within the Rasch measurement paradigm, which assumes that items with the same factor loading structure share identical factor loadings. This marks a key point of divergence from the 2PNO paradigm, where discrimination parameters are freely estimated. Investigating analogous models within the 2PNO paradigm, examining their properties, and generalizing the Volodin–Adams procedure to this paradigm represent promising directions for future research.

Finally, working in the confirmatory IRT paradigm, we do not discuss the consequences of the CORB model for the exploratory paradigm. Developing further rotation methods for the exploratory oblique bifactor analysis, determining the number of latent factors (Chen & Li, 2022), analyzing exploratory model fit, and other issues in the exploratory modeling also present a perspective avenue for further research.

Finally, since this paper operates within the confirmatory IRT paradigm, we do not discuss the implications of the CORB model for the exploratory paradigm. Developing advanced rotation methods for exploratory oblique bifactor analysis, determining the appropriate number of latent factors (Chen & Li, 2022), assessing exploratory model fit, and addressing other issues in exploratory modeling represent promising avenues for future research.

## Supporting information

Federiakin and Wilson supplementary material Federiakin and Wilson supplementary material

## Footnotes

1 More generally, they can be assigned to any real number values, as necessary for the Partial Credit Model.

2 We use the symbol  (solid vertical line) within square brackets to denote row-based matrix concatenation, ensuring clarity and avoiding confusion with matrix multiplication. Matrix multiplication is not possible in this context due to the non-conformable dimensions of the matrices involved. Similarly, Volodin and Adams (2002) addressed this potential ambiguity by using spacing between the symbols denoting matrices for the same purpose.

3 For simplicity, from this point forward, the model equations will be expressed in terms of proportionality functions to avoid specifying the full model denominator as in Equation 1.

## Supplementary material.

To view supplementary material for this article, please visit http://doi.org/10.1017/psy.2025.14.
