# Supplementary material for: Identification and Interpretation of the Completely Oblique Rasch Bifactor Model
Source: Psychometrika. 2025 Apr 24;90(4):1284–318. doi: 10.1017/psy.2025.14 (PMC12660021; doi:10.1017/psy.2025.14)
Supplement: Federiakin and Wilson supplementary material [file S0033312325000146sup001.zip › Suplementary-CORB.html]

The function for the Volodin-Adams procedure


# The function for the Volodin-Adams procedure

- 1 The code for the function
- 2 Example of the use
  - 2.1 The identified case
    - 2.1.1 Preparing of the
      arguments
    - 2.1.2 Running the function
  - 2.2 The unidentified case
    - 2.2.1 Preparation of the
      arguments
    - 2.2.2 Running the function
- 3 The contents of the resulting
  object

# 1 The code for the function

The function performs the Volodin-Adams procedure for dichotomous
tests. That is, it analytically (definitively) checks if the fully
oblique multidimensional Rasch model is identified.

```
Volodin_Adams_porcedure = function (sets, A, B) {
  if (length(sets)==ncol(B)) {
    AB = cbind(A,B)
    ABr = AB
    ABr = ABr[!apply(ABr, 1, function(row) all(row == rep(0, ncol(ABr)))),]
    for (r in length(sets):1) {
      rcd = sets[[r]][length(sets[[r]])]
      ccd = 1:ncol(ABr)
      ccd = ccd[ABr[rcd,] == -1]
      ccd = ccd[1]
      rck = sets[[r]][1:(length(sets[[r]])-1)]
      cck = NULL
      for (i in 1:length(rck)) {t = c(1:ncol(ABr))[ABr[rck[i],] == -1]
      cck[i] = t[1]
      remove(t)}
      ABr[rcd, cck] = 1
      ABr = ABr[,-ccd]}
    E = matrix(NA, ncol=ncol(B), nrow=ncol(B))
    for (c in 1:length(sets)) {
      for (r in 1:length(sets)) {
        E[r,c] = sum(ABr[sets[[r]],(ncol(ABr)-ncol(B)+c)])}}
    detE = det(E)
    rankABr = matrixcalc::matrix.rank(ABr)
    if (detE == 0) {c1 = "does not seem to be identified."
    c2 = " NOT "
    c5 = "NB!: Consider trying another sets of items. This set of sets of items might be just unfitting to show that the model is identified."} else {c1 = "is identified."
    c2 = " "
    c5 = " "}
    if ((rankABr == ncol(ABr)) & (rankABr <= nrow(ABr))) {c3 = "satisfied:"
    c4 = " == "} else {c3 = "UNsatisfied:"
    c4 = " != "}
    cat("The determinant of matrix E is ", detE,
        ", which means that the vector of shifting constants can contain", c2, "only zeros.", "\n",
        "Volodin-Adams' condition of", "\n", "rank(ABr) == D + Pr <= K - I", "\n", "is ", c3, "\n",
        rankABr, c4, ncol(B), " + ", (ncol(ABr)-ncol(B)), " <= ", nrow(A), " - ", (nrow(A)-nrow(ABr)), "\n",
        rankABr, c4, (ncol(B) + (ncol(ABr)-ncol(B))), " <= ", (nrow(A) - (nrow(A)-nrow(ABr))),
        "\n", "The model ", c1, "\n", c5, sep="")
    return(list("AB_original" = AB,
                "AB_reduced" = ABr,
                "E" = E,
                "Determinant of E" = det(E),
                "Rank of AB_reduced" = rankABr))
  } else {return("The number of item sets is not eual to the number of dimensions.")}}
```

# 2 Example of the use

## 2.1 The identified case

### 2.1.1 Preparing of the arguments

To run the function, you need to export the A and B matrices from TAM
package.

#### 2.1.1.1 Exporting A and B matrices

The simplest way to do so is to run a model with the desired
dimensional structure, with a single iteration, and export the desired
matrices.

Import packages and data.

```
library(TAM)
```

```
## Loading required package: CDM
```

```
## Loading required package: mvtnorm
```

```
## **********************************
## ** CDM 8.2-6 (2022-08-25 15:43:23)       
## ** Cognitive Diagnostic Models  **
## **********************************
```

```
## * TAM 4.1-4 (2022-08-28 16:03:54)
```

```
library(matrixcalc) # for the Volodin-Adams procedure

d = data.cdm03
```

Run a dummy-model to export the matrices.

```
m = tam.mml(d$data, Q=d$qmatrix[,2:6], control=list(maxiter=1, snodes=200, progress=F))
```

Render the matrices.

```
q = designMatrices(modeltype="PCM", maxKi=rep(1, ncol(d$data)), resp=d$data,
                   ndim=5, Q=d$qmatrix[,2:6], constraint="cases")
```

Object q contains all the needed matrices.

#### 2.1.1.2 Defining item sets

Then, you need to define the sets of items, from which one parameter
per set will be constrained to the negative sum of other parameters. The
sets are defined as a list. Since we want to use a model with 5
dimensions, we define 5 item sets:

```
sets = list(c(1,2), c(3,4), c(5,6),c(7,8), c(9:ncol(d$data)))
```

### 2.1.2 Running the function

The function will provide the user-friendly message regarding the
conclusion of the analysis.

```
result = Volodin_Adams_porcedure(sets = sets, A=q$flatA, B=q$flatB)
```

```
## The determinant of matrix E is -48, which means that the vector of shifting constants can contain only zeros.
## Volodin-Adams' condition of
## rank(ABr) == D + Pr <= K - I
## is satisfied:
## 16 == 5 + 11 <= 32 - 16
## 16 == 16 <= 16
## The model is identified.
##
```

## 2.2 The unidentified case

### 2.2.1 Preparation of the arguments

#### 2.2.1.1 Exporting A and B matrices

Define a random clear bifactor structure to show the output.

```
Q = matrix(0, ncol=3, nrow=ncol(d$data))
Q[,1] = Q[1:8,2] = Q[9:nrow(Q),3] = 1
Q
```

```
##       [,1] [,2] [,3]
##  [1,]    1    1    0
##  [2,]    1    1    0
##  [3,]    1    1    0
##  [4,]    1    1    0
##  [5,]    1    1    0
##  [6,]    1    1    0
##  [7,]    1    1    0
##  [8,]    1    1    0
##  [9,]    1    0    1
## [10,]    1    0    1
## [11,]    1    0    1
## [12,]    1    0    1
## [13,]    1    0    1
## [14,]    1    0    1
## [15,]    1    0    1
## [16,]    1    0    1
```

Run a dummy-model to export the matrices.

```
m2 = tam.mml(d$data, Q=Q, control=list(maxiter=1, snodes=200, progress=F))
```

Render the matrices.

```
q = designMatrices(modeltype="PCM", maxKi=rep(1, ncol(d$data)), resp=d$data,
                   ndim=3, Q=Q, constraint="cases")
```

#### 2.2.1.2 Defining item sets

Since this time we want to use a model with 3 dimensions, we define
only 3 item sets:

```
sets = list(c(1,2), c(3,4), c(5:ncol(d$data)))
```

### 2.2.2 Running the function

The function will provide the user-friendly message regarding the
conclusion of the analysis.

```
result = Volodin_Adams_porcedure(sets = sets, A=q$flatA, B=q$flatB)
```

```
## The determinant of matrix E is 0, which means that the vector of shifting constants can contain NOT only zeros.
## Volodin-Adams' condition of
## rank(ABr) == D + Pr <= K - I
## is UNsatisfied:
## 15 != 3 + 13 <= 32 - 16
## 15 != 16 <= 16
## The model does not seem to be identified.
## NB!: Consider trying another sets of items. This set of sets of items might be just unfitting to show that the model is identified.
```

# 3 The contents of the resulting object

The original A|B matrix with all parameters:

```
result$AB_original
```

```
##          I01 I02 I03 I04 I05 I06 I07 I08 I09 I10 I11 I12 I13 I14 I15 I16 Dim01
## I01.Cat0   0   0   0   0   0   0   0   0   0   0   0   0   0   0   0   0     0
## I01.Cat1  -1   0   0   0   0   0   0   0   0   0   0   0   0   0   0   0     1
## I02.Cat0   0   0   0   0   0   0   0   0   0   0   0   0   0   0   0   0     0
## I02.Cat1   0  -1   0   0   0   0   0   0   0   0   0   0   0   0   0   0     1
## I03.Cat0   0   0   0   0   0   0   0   0   0   0   0   0   0   0   0   0     0
## I03.Cat1   0   0  -1   0   0   0   0   0   0   0   0   0   0   0   0   0     1
## I04.Cat0   0   0   0   0   0   0   0   0   0   0   0   0   0   0   0   0     0
## I04.Cat1   0   0   0  -1   0   0   0   0   0   0   0   0   0   0   0   0     1
## I05.Cat0   0   0   0   0   0   0   0   0   0   0   0   0   0   0   0   0     0
## I05.Cat1   0   0   0   0  -1   0   0   0   0   0   0   0   0   0   0   0     1
## I06.Cat0   0   0   0   0   0   0   0   0   0   0   0   0   0   0   0   0     0
## I06.Cat1   0   0   0   0   0  -1   0   0   0   0   0   0   0   0   0   0     1
## I07.Cat0   0   0   0   0   0   0   0   0   0   0   0   0   0   0   0   0     0
## I07.Cat1   0   0   0   0   0   0  -1   0   0   0   0   0   0   0   0   0     1
## I08.Cat0   0   0   0   0   0   0   0   0   0   0   0   0   0   0   0   0     0
## I08.Cat1   0   0   0   0   0   0   0  -1   0   0   0   0   0   0   0   0     1
## I09.Cat0   0   0   0   0   0   0   0   0   0   0   0   0   0   0   0   0     0
## I09.Cat1   0   0   0   0   0   0   0   0  -1   0   0   0   0   0   0   0     1
## I10.Cat0   0   0   0   0   0   0   0   0   0   0   0   0   0   0   0   0     0
## I10.Cat1   0   0   0   0   0   0   0   0   0  -1   0   0   0   0   0   0     1
## I11.Cat0   0   0   0   0   0   0   0   0   0   0   0   0   0   0   0   0     0
## I11.Cat1   0   0   0   0   0   0   0   0   0   0  -1   0   0   0   0   0     1
## I12.Cat0   0   0   0   0   0   0   0   0   0   0   0   0   0   0   0   0     0
## I12.Cat1   0   0   0   0   0   0   0   0   0   0   0  -1   0   0   0   0     1
## I13.Cat0   0   0   0   0   0   0   0   0   0   0   0   0   0   0   0   0     0
## I13.Cat1   0   0   0   0   0   0   0   0   0   0   0   0  -1   0   0   0     1
## I14.Cat0   0   0   0   0   0   0   0   0   0   0   0   0   0   0   0   0     0
## I14.Cat1   0   0   0   0   0   0   0   0   0   0   0   0   0  -1   0   0     1
## I15.Cat0   0   0   0   0   0   0   0   0   0   0   0   0   0   0   0   0     0
## I15.Cat1   0   0   0   0   0   0   0   0   0   0   0   0   0   0  -1   0     1
## I16.Cat0   0   0   0   0   0   0   0   0   0   0   0   0   0   0   0   0     0
## I16.Cat1   0   0   0   0   0   0   0   0   0   0   0   0   0   0   0  -1     1
##          Dim02 Dim03
## I01.Cat0     0     0
## I01.Cat1     1     0
## I02.Cat0     0     0
## I02.Cat1     1     0
## I03.Cat0     0     0
## I03.Cat1     1     0
## I04.Cat0     0     0
## I04.Cat1     1     0
## I05.Cat0     0     0
## I05.Cat1     1     0
## I06.Cat0     0     0
## I06.Cat1     1     0
## I07.Cat0     0     0
## I07.Cat1     1     0
## I08.Cat0     0     0
## I08.Cat1     1     0
## I09.Cat0     0     0
## I09.Cat1     0     1
## I10.Cat0     0     0
## I10.Cat1     0     1
## I11.Cat0     0     0
## I11.Cat1     0     1
## I12.Cat0     0     0
## I12.Cat1     0     1
## I13.Cat0     0     0
## I13.Cat1     0     1
## I14.Cat0     0     0
## I14.Cat1     0     1
## I15.Cat0     0     0
## I15.Cat1     0     1
## I16.Cat0     0     0
## I16.Cat1     0     1
```

The reduced A|B matrix:

```
result$AB_reduced
```

```
##          I01 I03 I05 I06 I07 I08 I09 I10 I11 I12 I13 I14 I15 Dim01 Dim02 Dim03
## I01.Cat1  -1   0   0   0   0   0   0   0   0   0   0   0   0     1     1     0
## I02.Cat1   1   0   0   0   0   0   0   0   0   0   0   0   0     1     1     0
## I03.Cat1   0  -1   0   0   0   0   0   0   0   0   0   0   0     1     1     0
## I04.Cat1   0   1   0   0   0   0   0   0   0   0   0   0   0     1     1     0
## I05.Cat1   0   0  -1   0   0   0   0   0   0   0   0   0   0     1     1     0
## I06.Cat1   0   0   0  -1   0   0   0   0   0   0   0   0   0     1     1     0
## I07.Cat1   0   0   0   0  -1   0   0   0   0   0   0   0   0     1     1     0
## I08.Cat1   0   0   0   0   0  -1   0   0   0   0   0   0   0     1     1     0
## I09.Cat1   0   0   0   0   0   0  -1   0   0   0   0   0   0     1     0     1
## I10.Cat1   0   0   0   0   0   0   0  -1   0   0   0   0   0     1     0     1
## I11.Cat1   0   0   0   0   0   0   0   0  -1   0   0   0   0     1     0     1
## I12.Cat1   0   0   0   0   0   0   0   0   0  -1   0   0   0     1     0     1
## I13.Cat1   0   0   0   0   0   0   0   0   0   0  -1   0   0     1     0     1
## I14.Cat1   0   0   0   0   0   0   0   0   0   0   0  -1   0     1     0     1
## I15.Cat1   0   0   0   0   0   0   0   0   0   0   0   0  -1     1     0     1
## I16.Cat1   0   0   1   1   1   1   1   1   1   1   1   1   1     1     0     1
```

E matrix corresponding to the user-defined sets and the desired
dimensionality structure:

```
result$E
```

```
##      [,1] [,2] [,3]
## [1,]    2    2    0
## [2,]    2    2    0
## [3,]   12    4    8
```

The determinant of E matrix (is desired to be non-zero):

```
result$`Determinant of E`
```

```
## [1] 0
```

The rank of the reduced A|B matrix:

```
result$`Rank of AB_reduced`
```

```
## [1] 15
```
